# Exploring Co-creative Drawing Workflows

**DOI:** 10.3389/frobt.2021.577770

**Published:** 2021-05-14

**Authors:** Chipp Jansen, Elizabeth Sklar

**Affiliations:** ^1^Department of Engineering, Centre for Robotics Research, King's College London, London, United Kingdom; ^2^Lincoln Agri-Robotics, Lincoln Institute of Agri-Food Technology, University of Lincoln, Lincoln, United Kingdom

**Keywords:** human-robot interaction, drawing, user study, co-creative AI, collaborative AI, creative computing

## Abstract

This article presents the outcomes from a mixed-methods study of drawing practitioners (e.g., professional illustrators, fine artists, and art students) that was conducted in Autumn 2018 as a preliminary investigation for the development of a physical human-AI co-creative drawing system. The aim of the study was to discover possible roles that technology could play in observing, modeling, and possibly assisting an artist with their drawing. The study had three components: a paper survey of artists' drawing practises, technology usage and attitudes, video recorded drawing exercises and a follow-up semi-structured interview which included a co-design discussion on how AI might contribute to their drawing workflow. Key themes identified from the interviews were (1) drawing with physical mediums is a traditional and primary way of creation; (2) artists' views on AI varied, where co-creative AI is preferable to didactic AI; and (3) artists have a critical and skeptical view on the automation of creative work with AI. Participants' input provided the basis for the design and technical specifications of a co-creative drawing prototype, for which details are presented in this article. In addition, lessons learned from conducting the user study are presented with a reflection on future studies with drawing practitioners.

## 1. Introduction

Some art forms feature well-understood and well-explored traditions of collaboration, such as *improvisation* in music. Within drawing practise, collaborative or co-creative drawing is rare and less explored. The use of state-of-the-art technology to facilitate collaboration or inspire creativity has been considered in both music (e.g., Carot et al., 2020) and visual arts (e.g., Lewis, 2008; Kowaliw et al., 2012). At the same time, recent developments within and broader awareness of *Artificial Intelligence (AI)* have expanded the notion of human-computer co-generated creative content. In our work, we are interested in exploring how AI technology might contribute toward an artist's drawing workflow. Here, we present the results of a preliminary user study which we conducted in Autumn 2018 with two key objectives: (1) to gain understanding of drawing practitioners' current working environments and uses of technology, both in their everyday lives and in their art practice; and (2) to speculate with drawing practitioners on ways in which intelligent devices might collaborate with them, providing opportunities to enhance rather than detract from their art practice. Our analysis concentrates on identifying features and challenges to address in the next steps of our research programme: the development of a prototype system designed to enable AI-supported collaboration in co-creative drawing practice.

The aim of our preliminary user study was to be *exploratory*. Instead of relying on our own assumptions about how contemporary drawing practitioners work, we interrogated participants explicitly through survey questions and implicitly through observation of their drawing, captured on video, and provided them with an opportunity for reflection through a semi-structured interview. During the interview, we proposed to drawing practitioners the notion of a co-design space in which they could draw alongside an AI collaborator. These discussions led to a number of interesting observations and discovery of misconceptions, both on the part of the practitioners with respect to AI and on our part with respect to practitioners' willingness to consider human-computer co-generation of creative content.

Why do we believe that this study is of interest to the Robotic Arts community? In this community, people are concerned not only with robotic systems as art pieces—*artifacts*—but also with robotic systems that are part of the *process* of creating art. This user study is most applicable to the latter group, particularly with respect to human-robot interaction research (as elaborated in section 2 on Related Work). In our envisioned prototype system, our non-traditional “robot” is embodied within an intelligent system that assists a drawing artist in a variety of ways, as explored in this article. In this case, our system is learning through observation, i.e., learning by watching an artist draw. Eventually, through such learning and computational creativity models, robotic art systems might be able to embody more surprising and novel artistic styles. But, perhaps, the most novel outcome will be the collaborative relationship between a robot and their fellow artist.

This article is organized as follows: section 3 details the design of our user study, after which, the results are presented, split into two sections. Section 4 presents subjective analysis of results: how the participants described their drawing practice based on responses to our paper survey and discussions in the semi-structured interview. Section 5 presents objective analysis of results: we reviewed the video of participants drawing, captured during the sessions, and computed statistical metrics as well as considered image processing techniques to characterize aspects of their drawing process. Section 6 describes the next steps in our research programme: how we are currently applying the results from the study to the design and development of our prototype human-AI co-creative system. In section 2, we place our investigation into a wider context by highlighting pertinent related work. Key factors, identified during the user study, that influence the design of our prototype system are discussed in section 7. Finally, a summary of our study, reflection on lessons learned and plans for future work are shared in section 8.

## 2. Related Work

In this section, we look at works related to the topic of collaborative drawing between human artists and machines. First, we consider the studies of human artists and designers, either drawing solo (section 2.1), as well as recent research into drawing collaboratively (section 2.2). Then, section 2.3 contains a brief review of systems that generate drawings independently, either through bespoke programming, through rule-based systems or through learnt models. Both robotic drawing systems that draw physically and software-based digital drawing systems are considered. In section 2.4 we then review the state-of-the-art in co-creative drawing systems, where the drawing interaction is between humans and machines. Finally, section 2.5 identifies the gaps in the prior work that motivates the exploratory study.

### 2.1. Artists Drawing Alone

First we look at research on artists drawing by themselves and the technology used to study their practice. Video recording serves as the primary basis of early studies of drawing. Empirical psychological studies in the 1970's and 1980's involved hand annotation of videos showing the movement of an individual's hand and pencil to produce drawing (Van Sommers, 1984). The research output was a systematic catalog of features, such as the stroke order and preferential directions in mark-making between right-handed and left-handed individuals. The aim of these studies was to go from the mechanistic understanding of how drawings are produced to arrive at conclusions in how cognition plays a role in both representational and abstract drawing. The authors concluded that drawing was a vertical process, built up in layers, from drawn strokes, geometric primitives to conveying meaning through *graphic acts*. In addition, they conclude that artists are not conscious of the “structure and complexity of their own conduct” and requires a broad variety of analytical methods to understand drawing (Van Sommers, 1984, 270).

*Saliency analysis*, or analysing the movement of an individual's eye fixation to understand where the their attention lies, is another form of analysis used to understand drawing. One set of studies developed a gaze shift theory that describes the movement of an artist's attention between the canvas and the subject in observational drawing, with noted differences based on the artist's experience (Miall and Tchalenko, 2001; Tchalenko and Chris Miall, 2008; Tchalenko et al., 2014). Saliency analysis has also been used to try to understand eye fixation when drawing different categories of objects (Sarvadevabhatla et al., 2017).

Where eye tracking often requires wearing specialized sensors, other drawing studies approach observing an artist as unobtrusively as possible. A recent study of the painting process used a mixed-sensor approach comprising multiple cameras and microphones attached to the canvas (Fernando et al., 2018; Weiler, 2020). This research transforms the sensor data from the artist's drawing session into novel visualizations, such as time-lapsed video and 3D printed relief representation in order to provide the artist a means to reflect on the latent processes involved in completing the artwork.

### 2.2. Collaborative Drawing

*Design sketching*, or using sketching in the design process for architecture and engineering, has been studied as a communication tool between multiple human designers. In particular, comparing in-person human-to-human sketching to that of collaborative human-to-human remote sketching in terms of multimodal communication (Eris et al., 2014) has been studied. Studies also analysed the use of sketching and gesturing and compared how they differ when done in-person on a shared canvas to that on digital shared canvas (Zurita et al., 2008) or virtual environment (Gül and Maher, 2009). In design sketching, the sketch is not the final output. Instead it is a part of the process to achieve a design goal. In this regard, the sketching is different to that of illustration, where the drawing is the intended produced artifact.

Within the *drawing research* community (Garner, 2012), there has is an increasing interest in documenting the wide number of collective and collaborative drawing practises among artists (Journeaux and Gørrill, 2017). Often these practises involve drawing together either in person or via postal correspondence between two [e.g., the *Inappropriate Shift* project (Baker and Foster, 2017)] or a larger distributed group [e.g., the *Brew International Drawing Circles* (Brew and Journeaux, 2017)]. These collaborative practises are not technologically mediated, beyond coordination via e-mail or messaging application, and thus the drawing is analog in medium.

However, a very recent work (Parikh and Zitnick, 2020) in understanding human-human collaborative sketching takes the form of a crowd-sourced study. Using an online digital application, multiple individuals take turns sketching a scene with a finite stroke limit. In addition to taking turns, the individuals can vote on versions of the sketch to decide on which sketch will be used in the next round of collaboration. They found that this collaborative-voting strategy produced the highest perceived creativity in the results of their experiments. Also, unlike very personal relationship-based collaborative practices mentioned previously in the drawing research community, this collaboration is performed anonymously with individuals only communicating through what they draw and how they vote.

### 2.3. Robotic Systems Drawing Alone

Traditionally, human-robotic collaboration in the visual arts consisted of artists programming robots to draw imperatively. Perhaps the most classic robotic drawing systems is the *AARON* robot (McCorduck, 1991), which was programmed by the artist Harold Cohen to paint in a manner that he concluded was an extension of his own artistic style (Boden and Edmonds, 2019). Similarly, the portraiture robot, *PAUL* (Tresset and Fol Leymarie, 2013) drew real-life subjects via computer vision system. Like *AARON*, the style of the drawing was strongly influenced by its creator (Patrick Tresset). A contrasting approach toward drawing is relying on *emergence* and the complex interactions of a swarm of robots to produce a drawing, such as the *ArtSBot* and *Robot Action Painter (RAP)* systems (Moura, 2016).

An iconic project which embodies the spirit of constructing drawing systems is *The Painting Fool* system (Colton, 2012). While not a robotic system, it is a fully automated painter, which is software that simulates the behaviors of human painters. It makes choices about art materials and painting style and simulates strokes on canvases. In addition to producing work, it is a *Creative Computing (CC)* system which describes the output, through generating text and arguments about the produced work.

All of these systems incorporate a specific set of rules that drive the creation of the art. Some are large hand-developed systems of rules that were constructed over time, such as *AARON*. Others were a few simple rules, which relied on the complex interaction of many autonomous drawing robots to produce complex artworks, such as the *ArtSBot*.

In recent years, *Machine Learning (ML)* advancements in deep learning and neural networks and in particular the outputs from *Generative Adversarial Networks (GANs)* (Goodfellow et al., 2014) have produced a series of “AI Art” systems (McCormack et al., 2019), some of which were controversial by making large sales on the international art market (Unknown, 2018). These systems differ from the robot drawing systems mentioned previously in that they are entirely data-driven in how they produce the art work. An important distinction to make is that many of these systems are static raster-image based, often utilizing levels of *Convolutional Neural Networks (CNNs)* which produce pixel-level image data. *Style transfer* models are common systems here, which perform image-to-image translation. For example, the *pix2pix* system (Isola et al., 2017) is a style-transfer system capable of rendering a photograph into a painting of a specific artist's style. In terms of drawing systems, *SmartPaint* (Sun et al., 2019) is a painting style-transfer system that creates cartoon landscape paintings via a GAN trained on cartoon landscape images and their corresponding semantic label maps (i.e., images where color values correspond to semantic features). An artist sketches semantic maps, such as where trees and mountains are in a landscape, and the system generates a cartoon painting with appropriate colors and textures. While the authors describe human-machine co-creation in their paper, they also conclude that their machine would benefit from more human ownership in the design process as once they submit the semantic map the painting is generated entirely by the AI. An artist might feel more a part of the creative process if they were able to participate in the painting and creative transformation. to painting, would make the human feel more a part of the creative process.

The output of these models are the pixels of a completed image, where the robot drawing systems mentioned previously produce sequences of drawing actions. In this case, an early deep learning system (Graves, 2014) used *Recurrent Neural Networks (RNN)* to encode the drawing action sequences of doodles. This work was improved upon by the *sketch-rnn* system (Ha and Eck, 2017) which became a very influential neural network representation for sequences of drawing actions. This system uses a sequence-to-sequence *Variational Autoencoder* to encode sequences of drawn strokes learned from a large corpus of crowd-sourced sketches (Jongejan et al., 2016) and has inspired subsequent work in generalizing and modeling sketching (Chen et al., 2017; Cao et al., 2019; Lu et al., 2019).

### 2.4. Co-creative Drawing Systems

The *sketch-based* interaction research literature is rich with description of tools that provide real-time support to artists while drawing. These tools operate at a range of scales, from the drawing-primitive level, such as beautifying an artist's drawing with idealized geometric models (Arvo and Novins, 2000), to producing entirely new drawings, such as attempting to automatically draw the next frame in a drawn animation sequence (Xing et al., 2015). Alternatively, drawing systems can also support an artist by providing them with supportive imagery as an overlay or underlay to draw alongside. An example of this is the *ShadowDraw* system (Lee et al., 2011), which provides the artist with processed gradients from an object category database to draw over.

While there is interaction with the computer and the artist in these systems, there is not the encompassing goal to provide a truly co-creative experience between the artist and their drawing process. Co-creative systems expect the AI to be collaborating with the artist in the production of the artwork. An example of such a system is the *Drawing Apprentice* (Davis et al., 2016a,b), an improvising drawing agent that analyzes the user's input and responds with its own artistic contributions within a shared digital canvas. The AI can draw either by taking turns or asynchronously, and the artist has the ability to correct the AI as it is in the process of drawing. In addition, the Drawing Apprentice utilizes real-time object recognition on the artist's drawing as part of the inputs of the system. The artist is able to select drawing modes where the AI can trace, transform mimic the their drawn input.

The *sketch-rnn* model spurred the development of co-creative sketching systems. *collabdraw* (Fan et al., 2019) is a web-based collaborative sketching environment that uses the *sketch-rnn* model to allow an artificial agent to collaboratively sketch with a human with a well-defined visual concept from its sketch database (i.e., “Let's draw a bear together”). While the sketching goal was constrained, the authors were able to show in user studies that the collaborative sketches contained as much semantic content as those produced by the humans on their own. In *collabdraw*, the human and AI alternate strokes in a turn-by-turn manner. In contrast, an example of a continuous drawing system is *DuetDraw* (Oh et al., 2018). This system integrates *sketch-rnn*'s capabilities into a variety of tools that the AI can utilize with varying initiative in collaborative drawing. It can complete the artist's sketch, transform the sketch into a different style and recommends empty space on the drawing canvas for the artist to fill. In addition, it utilizes the *PaintsChainer* (Yonetsuji, 2017) style-transfer model in order to colorize a sketch. The drawing agent also communicates with the artist during the drawing process to explain why it is taking certain actions. *DuetDraw* is also an example of a system that fuses existing AI drawing models within the same interface.

The interaction between the artist and the AI does not need to involve the AI drawing directly on the art piece. The *Creative Sketching Partner (CSP)* (Karimi et al., 2019) is a co-creative design system that collaborates with a designer on a shared design task. In order to prevent design fixation, the CSP uses *conceptual shifts*—a conceptual model that guides users toward different aspects of a design space based on visual and conceptual/semantic similarity. Utilizing the *QuickDraw* dataset (Jongejan et al., 2016) as a database of sketch designs that can be related to the working sketch both visually and semantically, it present the designer with a novel sketch that is visually similar but semantically different.

Co-creative collaboration can also occur beyond the digital canvas but in physical space. The *DialogCanvasMachine* (Cabannes et al., 2019) was a physical interactive installation involving an artist duo, Tina&Charly, who already have a collaborative painting practise, and an AI using the *sketch-rnn* model trained on the *QuickDraw* dataset and a catalog of the artists' work. The three collaborators take turns painting on a canvas in their own unique color. During the AI's turn, it takes a picture of the canvas and responds by projecting suggested strokes onto the canvas, which are interpreted by the artists and painted onto the canvas. Like the *Drawing Apprentice*, the artists have the ability to interpret and edit the contribution of the AI to the painting.

Human-robotic collaborative drawing is another example of art-making in physical space. The *D.O.U.G* system (Chung, 2015) involves an industrial robot collaborating with the artist Sougwen Chung to produce paintings. The robot is programmed to mimic what the artist is drawing and in turn the artist can respond to what the robot is drawing (Sandry, 2017). This occurs upon the same canvas in a real-time continuous manner. Another collaborative robot painting project is the *ArtTherapyRobot* (Cooney and Menezes, 2018; Cooney and Berck, 2019). This system's goal is to conduct research into socially assistive robotics for art therapy. Using a Baxter^1^ robot, the system and the artist paint separate pieces or take turns painting on the same canvas. The robot operates in two painting modes, one where it imitates the artist's painting. In the mode it attempts to sense the emotional state of the artist and contributes to the painting according the a visual metaphor model.

### 2.5. Conclusions

Our search of the literature with respect to co-creative or collaborative drawing amongst humans and machines was broad. The related work shows that there is a history of studying artist's drawing through technology (section 2.1), a body of research into humans drawing collaboratively (section 2.2), a rich history of computational creative systems drawing autonomously (section 2.3), as well as recent research into co-creative drawing systems (section 2.4). Three motivations for our user study arose from the related work.

Previous research appears to assume that co-creative drawing is a mode well-understood by practicing artists. While there are studies into artist's drawing behavior (section 2.1) and human-human collaborative drawing (section 2.2), we are unaware of any study that discusses directly with practicing artists their attitudes toward and the opportunities for co-creative drawing systems. Thus, our preliminary survey was motivated by a desire to explore these specific questions with practicing artists and pose direct questions to them about working with an AI-driven assistant.

In section 2.4, we see that some co-creative systems are evaluated through user studies, such as testing *conceptual shift* techniques with the *CSP* (Karimi et al., 2019). However, we are not aware of any preliminary study involving practising artists contributing toward the design of a co-creative drawing system. Systems, such as the *DialogCanvasMachine* (Cabannes et al., 2019) or *D.O.U.G*. (Chung, 2015) were developed in conjunction with a single artist or artist duo. Or, system designers were themselves artists who developed a suite of co-creative drawing systems, such as the *Drawing Apprentice* (Davis et al., 2016a). We see our preliminary study as an opportunity to inquiry multiple and possibly contradicting perspectives onto the possibilities of co-creation between a human and an artist.

Finally, robotics and sensor fusion provide opportunities for co-creative systems to operate with artists working with physical mediums. The *ArtTherapyRobot* (Cooney and Menezes, 2018), *D.O.U.G*. (Chung, 2015) and the *DialogCanvasMachine* (Cabannes et al., 2019) are inspiring examples of co-creation in physical space. Given the dominance of digital drawing tools within an artist's practise, we wanted to use the preliminary study to discuss the use of physical mediums and the opportunities for co-creative drawing with them.

## 3. Design of Preliminary User Study

This section describes the design of our preliminary user study which we conducted in Autumn 2018. The objectives of the study was to improve our understanding of drawing practitioners' working environments and their views toward a future system in which they could draw with an intelligent “robotic” collaborator. Recruitment for the study was done via email solicitation to a university research study recruitment channel, art schools and London based drawing communities. Respondents were filtered to balance 3 classes of participants: part-time drawing enthusiasts, full-time professional illustrators and full-time illustration students. In total, 21 participants were interviewed individually for 90–180 min sessions each consisting of three activities: a paper survey (section 3.3), a series of video-recorded drawing exercises (section 3.4) and a semi-structured post-interview (section 3.5).

### 3.1. Ethical Clearance

This study was carried out in accordance with the recommendations of the Research Ethics Office of King's College London as Low Risk Research, approved by the university's Biomedical & Health Sciences, Dentistry, Medicine and Natural & Mathematical Sciences Research Ethics Subcommittee. All subjects gave written informed consent in accordance with the Declaration of Helsinki.

### 3.2. Demographics

**Figure 1:**
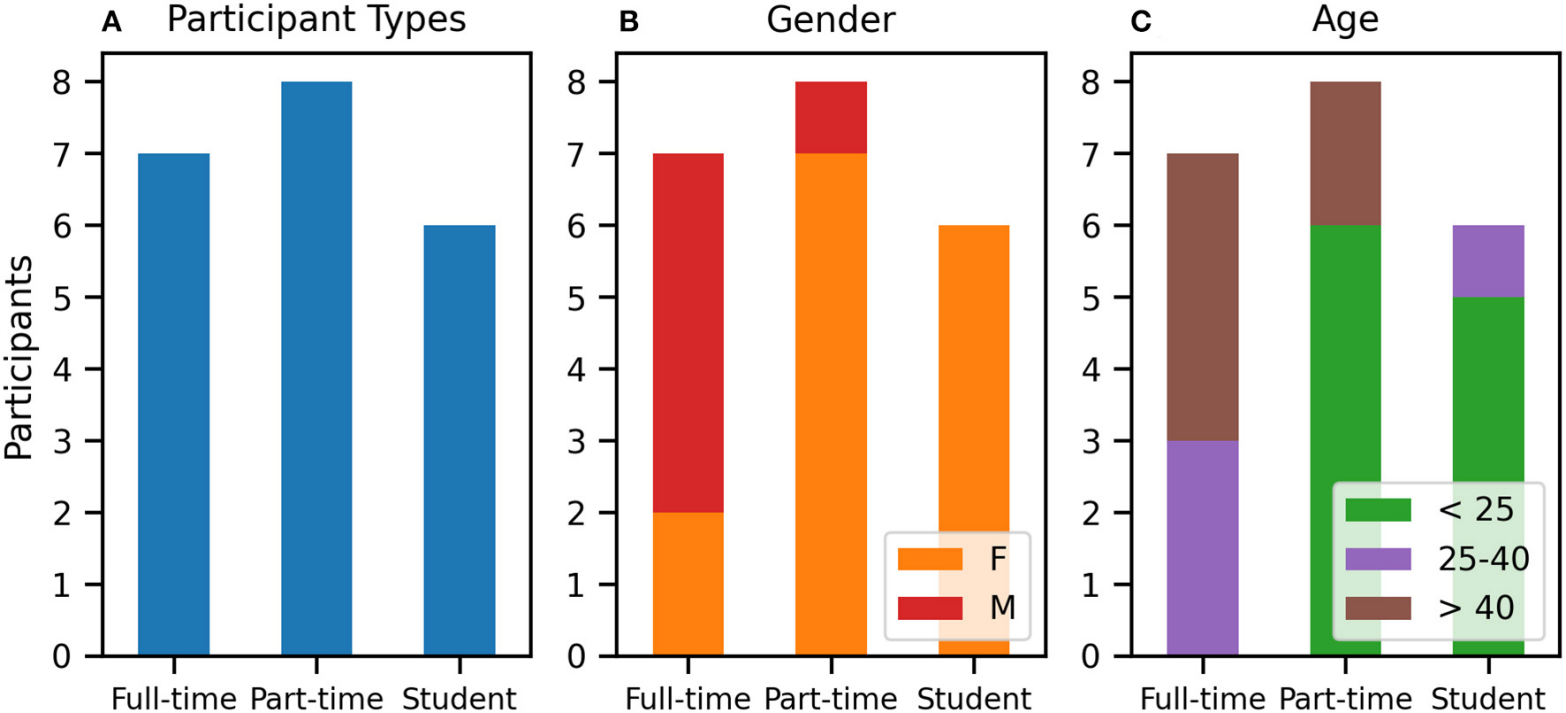
Study participants, showing **(A)** drawing practitioner types, **(B)** gender, and **(C)** age distributions.

The study had 21 participants, representing a balanced mix of three groups: professional illustrators (*n* = 7), part-time drawing enthusiasts (*n* = 8) and illustration students (*n* = 6). Gender-wise, the group skewed two-thirds female. However, a majority of full-time artists are male. Age-wise, the majority of students and part-time drawing practitioners represented the younger (<25) group, whereas the full-time artists were spread across the older groups (25–40 and > 40). Figure 1 illustrates the demographics.

### 3.3. Paper Survey

As the first activity, each participant completed a 21-question paper survey consisting of multiple choice and free response questions about their drawing habits, technology usages and attitudes. The survey was divided into three sections: Background, Drawing Practise, and Technology (usage and attitudes). Table 1 contains the text of each question, identified as **Q1** through **Q21**. A copy of the survey is available in the Supplementary Material. The survey elicited four different types of responses: (a) multiple choice only; (b) multiple choice with open-ended “other” option; (c) open-ended only; and (d) positive scale (i.e., 0..*N*).

**Table 1:** Survey questions, with reference to the section in which the responses are discussed.

	**Question (clarifications given in italics)**	**Related section (§)**
**Q1**	How long have you been drawing as part of your creative or professional practise?	Experience (§ 4.1.1)
**Q2**	Have you done any formal training for drawing?	Experience (§ 4.1.1)
**Q3**	Do you participate in any drawing communities, collectives, groups, or drawing sessions (i.e., life drawing sessions)?	Drawing Prac. (§ 4.1.2)
**Q4**	Do you earn money from your drawing practise or do you utilize drawing as part of your profession?	Drawing Prac. (§ 4.1.2)
**Q5**	Rate the extent to which you would describe your drawing as being.	*n/a*
**a**	.illustrative?	
**b**	.abstract?	
**c**	.drawn from real-life? (rendering an object, setting or subject that you directly perceive)	
**d**	.drawn from personal memory? (rendering an object, setting or subject that you perceived in the past)	
**e**	.drawn imagination?	
**f**	.an expression of emotion?	
**Q6**	How frequently do you doodle or make drawings while your attention is otherwise occupied (i.e., draw absentmindedly during a meeting in the margins of a piece of paper)?	*n/a*
**Q7**	Which drawing mediums do you most typically draw with?	Media&Tech. (§ 4.1.3)
**Q8**	When do you draw? Is there a regular time (i.e., in the morning, late at night) or routine (i.e., with a coffee, after a long walk) that you have with your drawing practise?	*n/a*
**Q9**	How long are your drawing sessions typically?	Timing (§ 4.1.4)
**Q10**	How do you typically work? (*How often do you take a break while working?*)	Timing (§ 4.1.4)
**Q11**	How do you typically focus your work during your drawing sessions?	Timing (§ 4.1.4)
**Q12**	What is your drawing environment like?	Environment (§ 4.1.5)
**Q13**	Are there things about your drawing environment that you would want to change to make it a more ideal work setting?	Environment (§ 4.1.5)
**Q14**	Do you carry around a sketchbook or a portable drawing pad?	Media&Tech. (§ 4.1.3)
**Q15**	Do you practise collaborative or collective drawing with another person or persons?	Drawing Prac. (§ 4.1.2)
**Q16**	Which of the following technologies do you utilize on a regular basis?	*n/a*
**Q17**	Which of the following technologies do you utilize as part of your drawing practise?	Media&Tech. (§ 4.1.3)
**Q18**	Which of the following technologies do you utilize to capture, document, or archive your drawn work?	Media&Tech. (§ 4.1.3)
**Q19**	Which of the following ways do you use to share, distribute, or sell your drawings?	*n/a*
**Q20**	How interested would you be to utilize more technology in your drawing practise?	Interest (§ 4.1.6)
**Q21**	Is there anything else that you would like to say or comment on?	*n/a*

*Questions with related section marked n/a are not explicitly analyzed in this article as the results were not deemed useful with respect to the explicit objectives laid out in section 1. Question numbers are color coded: (red) multiple choice only; (orange) multiple choice with open-ended “other” option; (yellow) open-ended only; and (blue) positive scale (i.e., 0…N). The full survey for the study is available in the Supplementary Material*.

In section 4.1, we analyse the 15 questions that contributed most to our objectives: gaining understanding of drawing artists' current working environments and technology usage and speculating on ways in which intelligent devices might collaborate with drawing artists to enhance their practice. Other questions (**Q5, Q6, Q8, Q16, Q19, Q21**) queried broader baseline aspects of the participants' drawing practise and attitudes toward technology. In the end, we did not find them pertinent as contributing to the design of a co-creative system and did not include analysis of these questions in this paper.

### 3.4. Drawing Exercises

The second activity was a series of three video-recorded exercises, each lasting ~10 min. Time limits were necessary to implement due to practical factors around scheduling of study participants. In practice, professional drawing may be constrained by deadlines (e.g., publication or exhibit schedule) or more open-ended (e.g., fine art). Artists may behave differently depending on what motivates their drawing, so we designed the study to prompt three different “types” of drawing. Participants drew from *observation*, from *recollection* and from *imagination*, as explained below. The order of the exercises was the same for each participant. The post-interview (described in section 3.5) captured artists' impressions of these different exercises and how they relate to their own practice (see section 4.2.1. The prompts given to the participants were as follows:

- **Observation**: *Here is an arrangement of objects on the table for you to draw, however you feel fit*.
- **Recollection**: *Next, from memory without any reference material, draw a bicycle, or bicycles*.
- **Imagination**: *Finally, do some free drawing, which could be anything, real or imaginary. It could be many things, or one specific thing. Draw anything*.

For exercise A (Observation), they were presented with a small set of objects (e.g., coffee cup, small figurines, and plastic fruit). The layout and selection of props varied depending on where the research study setting. Most of the study sessions (*n* = 16) occurred in a studio-like setting in the Interaction Lab at King's College London, while the others took place in participants' studios (*n* = 5). An assortment of drawing tools were available to the participants (see Table 2): pencils (various weights and colors), charcoal, pens (various line weights) and graphics markers. In addition, participants were encouraged to bring and use their own personal drawing tools. Results are analysed in section 5.

**Table 2:** Drawing tool usage.

**Tool**	**Number of artists**
Charcoal	4 (19%)
Eraser	**12** (57%)
Finger smudge	2 (10%)
Graphite	2 (10%)
Marker	7 (33%)
Pastels	1 (5%)
Pen	**12** (57%)
Pencil	**17** (81%)
Stylus	1 (5%)
Watercolors	1 (5%)

*Bold indicates most common tools used by participants*.

### 3.5. Post-interview

The final activity was a semi-structured interview in which we asked a few open-ended questions about participants' drawing practice and discussed follow-up questions about the drawing exercises. In addition, we asked them about their attitudes toward AI and envisioning potential collaboration with a drawing AI. We utilized three prepared questions to spur discussion, initiated by each of the prompts listed below:

1. **Drawing Practice**: *Reflect on the drawing session, how does this compare to how you use drawing in your work and part of your creation process?*
2. **Attitude toward AI**: *I [the interviewer] am interested in collecting people's viewpoints about what they perceive about Artificial Intelligence (AI). The term AI has changed a lot in the media in popular usage and culture over the years since when I studied it. For example, at one point graphical user interfaces were considered AI because of the novelty of using a visual interface to interact with a computer. What does AI mean to you?*
3. **Co-design Question**: *I [the interviewer] am interested in developing a technical tool that artists can collaborate with in their drawing practise. While there is a definitive digital practise with drawing, I am investigating whether there is still value in having artists working and drawing with traditional media (i.e., pen and ink on paper). I envision this artist tool as observing and responding to what the artist is drawing on the page in a real-time process. Such a tool could be a form of sketchbook that has a sense of what you have drawn before, or an improvising partner in collaborative or collective sketching. Based on your experiences with drawing, and your drawing practise, I'm interested in hearing your reaction and ideas around such a system*.

Results are analysed in section 4.2.

## 4. Subjective Results: Surveys and Interviews

In this section, we discuss the results and subjective analysis of the first and last components of the study: answers to survey questions and feedback obtained during the semi-structured interview.

### 4.1. Survey Results

Capturing information about the drawing tools and technology usage of participants is key for understanding the range of physical art media that drawing artists employ and thus highlight potentially important features of our prototype co-creative drawing system. Since the aim of our survey was exploratory, some questions proved to be more relevant than others for the purposes of guiding the design of our prototype. Here we focus on the questions (listed in Table 1) which provided more pertinent answers, addressed accordingly in the following sub-sections.

#### 4.1.1. Experience and Education

**Figure 2:**
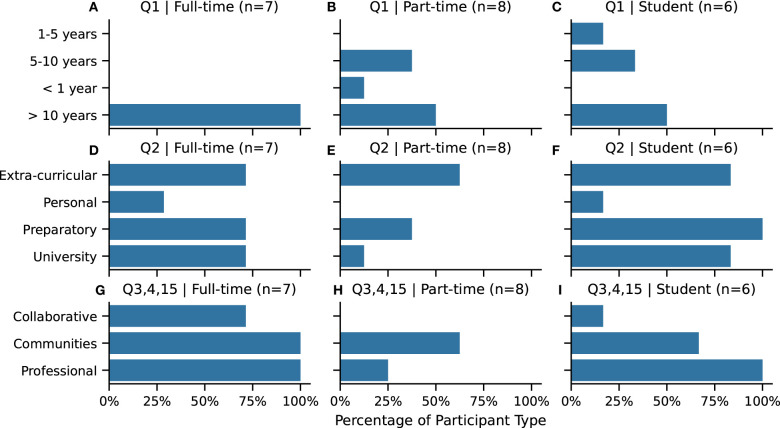
**(A–C)** Q1. Self-reported years spent drawing. **(D–F)** Q2. Drawing education. **(G–I)** Drawing practise: participation in **collaborative** or collective drawing (Q3), in drawing **communities** (Q4) or as a **professional** drawer (Q15). Abbreviations are in **bold**. Participants selected all answers that apply, except for Q1 where participants selected only one category.

With regard to experience, all of the full-time artists and half of the part-time artists and student participants reported to have been drawing for longer than 10 years (Figure 2, **Q1**). Most participants have had some formal drawing education (Figure 2, **Q2**). Both the full-time artists and the student participants had preparatory (A-level or foundation level) drawing instruction or some form of university drawing training. Extra-curricular drawing training, such as workshops or continuing education drawing courses, were the most common form of training for part-time participants. Less common across all of the participant types were personalized modes of drawing training (e.g., one-on-one tutorials or apprenticeships).

#### 4.1.2. Drawing Practices

Participation in drawing communities was most reported by all full-time artists, and over half of the part-time artists and students (Figure 2, **Q3**). The most common kinds of drawing communities people participate in are regular life drawing courses and urban sketching meet-ups.

All of the full-time artists and students reported making money with drawing (Figure 2, **Q4**). Professional activities described were illustration (full-time and freelance), selling artwork at art fairs and online and doing bespoke commissions, such as portraits or commercial sign painting.

Collaborative or collective drawing activity was nearly non-existent for part-time artists and students (Figure 2, **Q15**). But a majority of full-time participants have reported to have had some experience. How the participants interpreted collaborative or collective drawing varied (as there was no definition given on the survey, see **Q15** in Table 1). Example interpretations for collaborative drawing included sharing drawings with each other at life drawing classes or drawing on each other's canvases in a round-robin style for limited periods of time.

#### 4.1.3. Mediums, Tools, and Technologies Used to Draw

Our survey asked about the kinds of mediums artists use for drawing, either *analog* (drawing with physical media) or *digital*, as these answers help inform the technical design of our prototype system and assess the novelty of our approach.

**Figure 3:**
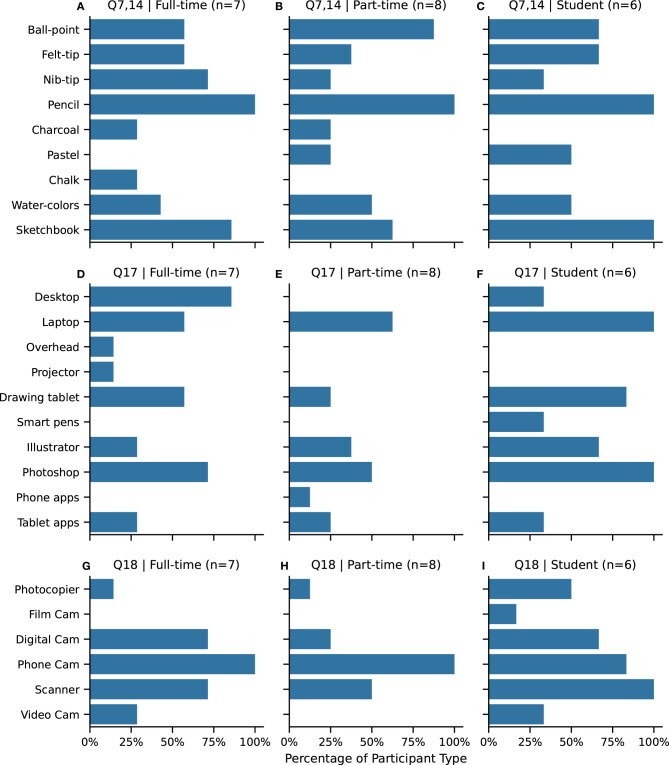
**(A–C)** Q7. Drawing materials, including pens (**ball-point**, **felt-tip**, **nib-tip**) and Q14. **sketchbook** usage. **(D–F)** Q17. Technology used for drawing, including **desktop** computers, **laptop** computers, analog **overhead** projectors, digital **projectors**, Adobe **Illustrator**, Adobe **Photoshop**, mobile **phone** drawing **app**lications, **tablet** (e.g., iPad) drawing **app**lications. **(G–I)** Q18. Drawing capture technology, including analog **film cam**era, **digital cam**era, mobile **phone cam**era, digital **scanner**, digital **video cam**era. Abbreviations are in **bold**. Participants selected all answers that apply for all questions.

Pencil is universally used by all research participants, with pen and ink and water-color being common types of analog media (Figure 3, **Q7**). In addition, sketchbooks are used by a majority of the participants (**Q14**). All of the student participants use sketchbooks, as maintaining a sketchbook is a common activity as part of their drawing curriculum.

With regard to drawing technology (Figure 3, **Q17**), a laptop is more common than a desktop, although a desktop is used exclusively by full-time artists and some students. This might indicate a more dedicated work-space or use of university facilities. A specialized drawing tablet is the most common drawing interface. Whereas, tablets, smart pens and mobile phone drawing apps are less common. Software-wise, Adobe Photoshop is more commonly used than Adobe Illustrator. Only one participant uses a projector while working, which means interacting with projected imagery is rare within the study group.

With regard to digital capture technology (Figure 3, **Q18**), a mobile phone camera is the most common way to convert an analog drawn image into a digital form. Digital scanners are commonly used as well, in particular amongst student participants.

#### 4.1.4. Timing

**Figure 4:**
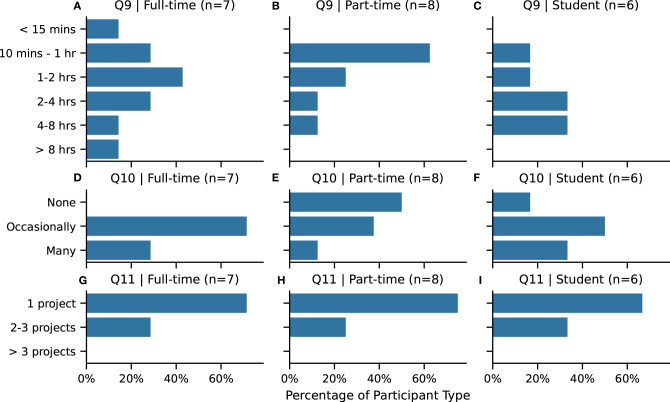
**(A–C)** Q9. Duration of drawing session. **(D–F)** Q10. Break frequency while drawing: **none** or uninterrupted no breaks, **occasionally** take a break, take **many** breaks. **(G–I)** Q11. Project focus while working: focus on **1** or single project, switch between **2–3 projects**, switch between **more than three projects**. Abbreviations are in **bold**. Participants selected one answer for each question.

How long and how often participants spend time working informs aspects of the technical specification of our prototype system. We want to ensure that the system is practically able to record all of the data generated while an artist is drawing. The participants were asked how long they typically spend drawing in a session (Figure 4, **Q9**). Where a majority of part-time participants spend 10 min to 1 h, both full-time artists and students reported longer drawing sessions.

They were also asked to assess how often they take breaks (Figure 4, **Q10**), where “occasionally take a break” was the most common response amongst full-time artists and students, while part-time artists leaned more toward “uninterrupted no breaks” response. With regard to switching context between projects during a working session (Figure 4, **Q11**), a majority of all participants focus on working on a single project at a time.

#### 4.1.5. Work Environment

**Figure 5:**
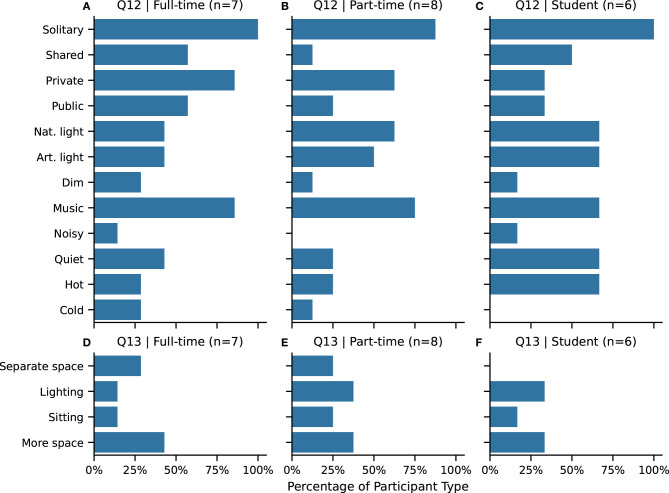
**(A–C)** Q12. Characteristics of work environment: **solitary** work environment, **shared** work space, **private** setting, **public** setting, **nat**urally bright environment, **art**ificial bright environment, **dim**ly lit environment, listening to **music**, **noisy** environment, *quiet* environment, **hot** (temperature) environment, **cold** (temperature) environment. **(D–F)** Q13. Improvements to the work environment (annotated from the Q13 free responses shown in Table 3): dedicated or **separate** studio space, improved **lighting**, improved **sitting**, **more space** or table space. Abbreviations are in **bold**. Participants selected any combination of answers.

Participants were asked about various physical characteristics of their work environment (Figure 5, **Q12**), as we are interested in understanding how participants work with respect to different types of distractions within their work setting. Depending on the level of environmental distraction, a collaborative drawing system may have to compete with noise levels, for instance, which could impact design decisions around providing audio feedback to users.

A solitary work environment was the most common response across all participants. In addition, a little over half of the full-time artists and students responded that they also work in shared spaces. Less than 25% part-time artists work in a shared work space. Private settings are more common than public work settings, overall across all the participant categories.

Half of full-time artists and students and a quarter of part-time artists reported that their work environment was quiet, and almost none of the participants reported working in a noisy environment. Listening to music was one of the most common responses across all participant types.

Bright environments were more commonly reported than dim environments. Overall, there was little distinction between natural and artificial lighting.

Because one's current work environment is often not the *ideal* work environment, participants were also asked about things that they would want to change to make improve their work setting (**Q13** in Table 1). A list of their responses are in Table 3. We found common themes within the free-responses and encoded them as:

- Having a dedicated, private or separate studio space to work in (*n* = 4).
- Improved lighting or better access to natural light (*n* = 6).
- Improved seating (*n* = 4).
- Having more space to spread out the work (*n* = 8).

Figure 5 shows the annotated counts broken down by participant type.

**Table 3:** **Q13** Responses to “Are there things about your drawing environment that you would want to change to make it a more ideal work setting?”.

**Type**	**Things to change about drawing environment (Q13)**
Full-time	In terms of comfort, I'd perhaps like a better sofa.
Full-time	One could always use more storage space, work space etc. A quiet space is ideal, but with people coming and going nearby, so as not to feel isolated. I would also like more space for printmaking (linocut etc).
Full-time	I would like to have a private studio.
Full-time	Yes! Would love to have a dedicated “At home” studio space with large longer desk 2–3 m long under a large long window surrounded by plants and good coffee please.
Full-time	Would like to work at home/studio more often!
Full-time	Currently I work from home. It would be nice to be able to make more of a mess.
Full-time	No.
Part-time	More natural light and more nature (but, being in London …)
Part-time	I like drawing anywhere quiet with good light, but perhaps it would be nice to have a proper studio space.
Part-time	I prefer natural light—sitting by windows—with enough space around me to layout my supplies. I usually put on music or background YouTube videos while drawing.
Part-time	Yes, ideally it would be a separate room where I only draw or paint
Part-time	No
Part-time	Better lighting, a larger surface I can spread my equipment on.
Part-time	A better chair so that I can improve my posture. I am also looking to get a larger table as well as —— in great —, such as felt-tips.
Student	Brighter light in my room when it's night.
Student	In my studio in university, I wish I had a little more space, as I do like to spread. At my student house, I wish I had more natural lighting as its a very dark house with only one window (studio apartment)
Student	I think its ideal enough.
Student	Add things for comfort—i.e., a chair with a back so my back doesn't hurt leaning over or footstools.
Student	I'd like more wall space. But I'd also like more people to be around working as well.

#### 4.1.6. Interest in Using More Technology

**Figure 6:**
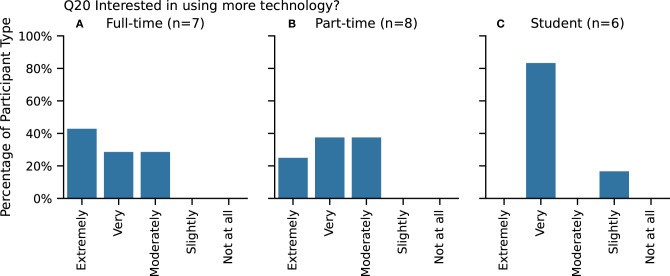
Interest in using more technology for **(A)** Full-time, **(B)** Part-time, and **(C)** Student participants. Participants selected only one answer.

With regard to interest in utilizing more technology in their drawing practice, over two-thirds of each participants group were either “Very” or “Extremely enthusiastic” (Figure 6, **Q20**), with the student participants being the highest majority.

#### 4.1.7. Analysis and Discussion

Overall, the surveys contributed a number of findings related to the working habits of our participant group. First, we wanted to verify that certain expected characteristics of different types of drawing practitioners held true. For example: the full-time artists and students draw more than the part-time artists. The full-time artists (and students) make money from their drawing activities, whereas this was less the case for the part-time artists. Second, the survey results provided information about and a distribution for the types of materials that these artists work with, such as pencil being a very common drawing medium. Third, the survey provides an indication for how long a typical drawing session will last, i.e., ranging from 10 min to 1 h, and focus on a single project being the most common activity. Fourth, we are better informed regarding the drawing software and hardware devices participants use, with Photoshop and drawing tablets being very common. Finally, the survey served to prime the interview at the end of the study session. Having the survey answers available during the interview allowed the interviewer to hone in on particular workflows that a participant might follow in creating their artworks.

### 4.2. Interviews

Each study session began with participants completing the survey on paper, discussed above; followed by executing three drawing exercises (described in section 5) and concluded with a semi-structured one-on-one interview with the first author of this article. Although these sessions were intended to be exploratory, as a guideline we utilized three main prompts (described in section 3.5) to cover the topics of our study. Section 4.2.1 contains analysis of answers to the first prompt, how the participants use drawing in their practise.

The second and third prompts from the interviews focused on participants' attitudes toward AI and then their reaction to the proposal of collaborative drawing with an AI. The participants had a range of attitudes toward AI. However, these attitudes came out more distinctly when considering having an AI drawing partner. Thus, for the purposes of this article, we will focus on the discussions that came out of the reactions to considering what forms AI might take as a co-creative partner, which we discuss in section 4.2.2.

#### 4.2.1. Drawing Practices

The first prompt of the interview explored how participants use drawing. During the interviews, the participants revealed many purposes for their drawing: from commercial illustration, to satisfying the requirements of design school projects, to life drawing in community classes.

While the discussion was initiated by asking them to compare the drawing exercises to how they use drawing in their regular practise, it often led to discussing particular projects and their workflow within those projects. The discussion included what types of drawing activity they do, with what kinds of materials and what kinds of workflows with technology do they utilize in their drawing. This was, by far, the largest and most free-flowing part of the interview process, often going off on long tangents. The aim was to spend time with them discussing their drawing practise without a specific structured flow to the conversation. As part of this process, some participants shared images of their work, or brought sketchbooks or examples of their work to the interview. Some also brought images of their work environment, or demonstrated some drawing techniques that they utilize as part of this portion of the interview. Overall, general patterns of drawing practises vary by participant type.

Part-time artists often do life drawing, or portraiture, usually in a group or life-drawing session. Subjects for their portraits often were their friends or family members. Character drawing, either celebrities or “fan-fiction” style drawing (i.e., their favorite anime characters) was another common practise. It is common for part-time artists to have done a high-school level focus on fine art and illustration. Some part-time artists had an education background in fine art, but decided on other primary career paths. Finally, a few of the part-time artists make money through selling their art works, often in online market places (e.g., Etsy^2^).

Students' drawing practices are primarily consumed by university coursework, although it is very common for them to distinguish between what they are drawing for university compared to what they are drawing for personal or freelance side-projects. The university coursework primarily consists of illustration studio modules for print production or screen. A few of the participants use drawing as part of design studios, such as logo design. *Research drawing* is a common practise, where the students draw from still-life (e.g., the zoo), online images or videos. Another type of drawing that was distinguished is *editing drawing*, or making multiple iterated revisions of a drawing to achieve a final product. One common practise for students is to maintain a project sketchbook, which consists of research drawings, planning and integrating other source media (e.g., cut-outs of images from the internet). The sketchbook is almost always a physical artifact. Students aspire toward careers with drawing. Very common professional aspirations include children's book illustrator, a profession that relies heavily on varied textured illustration (often quite analog or physical). Illustrating graphic novels or comics is another very common aspiration, as is drawing for animation or movies.

Full-time artists described drawing practices more closely to direction given by a client in the form of a brief or a commission. Briefs are common in editorial illustration, providing illustration for a publication, magazine or web-site. In this case, a client might have very vague ideas of what they want and are hiring the participant to contribute creative input. Commissioned work is typically representational drawing: human or pet portraiture from an image or a sitting or architectural drawing of someone's vacation cottage. Other paid work includes drawing in the built environment, either painting murals, sign painting or other text, such as menus for pubs. Another form of paid work is to be a live scribe, that is someone who visually annotates (usually on a white board) a live event, such as a lecture event or a meeting to provide a visual map of images and words as documentation.

Beyond commissions, professionals also generate imagery that they can sell as individual pieces. Often these are prints of illustrations, sold online and at art fairs. Often the original creative work advertises the artist and generates future commissions. Non-paid drawing activity for the full-time artists often consists of participating in drawing communities, in the form of life-drawing classes or urban sketching meet-ups. Personal work is rare, and often it is work with the aim of developing a professional project, such as pitching a storyboard for a movie production. None of the full-time artists are professional studio artists, who typically make a living through the sale of their work through a commercial gallery.

The primary content that all of the participants drew was either observational or illustrative, and most did not mention self-expressive or “free” drawing as being a part of their practice. Painting expressively and channeling their emotional state was mentioned by only one participant. However, *doodling* (i.e., free drawing in the margins of a notebook during lectures or meetings) is an extremely common practise according to question **Q6** in the survey.

Finally, regarding documenting and distributing their work, it was very common for many of the participants to post their work on social media (e.g., Instagram^3^). However, none of them live-streamed or produced videos of their work or process.

#### 4.2.2. Co-designing the Co-creative

This section contains analysis of answers to the third part of the interview, where we discussed the prospect of a co-creative drawing system and how it might have utility in their drawing practise. Here, we are interested in their views on the idea of drawing activity that occurs with an AI observing, processing and/or contributing to artwork in real-time.

As the last question of the interview, a description of the idea of a co-creative drawing system was given. They were asked about their initial reaction and what utility it might contribute to their drawing practise. In addition, they were asked to contribute ideas how it might be useful in their drawing practise. This was to get a response to the proposal of a collaborative drawing AI as well as to brainstorm about what the AI could do or offer to an artist.

In the survey, very few of the participants (*n* = 6, see section 4.1.2) reported to having participated in any collective or co-creative drawing, technology-mediated or otherwise. For most of the participants, they first had to consider what forms of collaborative drawing could take place, in general, regardless of whether it was with an AI or another person.

Based on this, common initial reactions to the question placed the AI in the role of a tutor or fellow peer at a life drawing class—i.e., substituting for human roles that the participants are familiar with. In these situations, the AI would observe the drawing and offer corrections or critiques of the drawing artifact or process. This implies that the AI has some notion, or assumption, as to what the artist is attempting to draw and the “correct” way of drawing or rendering of that image. In the case that the artist is drawing from observation, the AI might also observe the world or still-life being rendered. Alternatively, if the drawing is not from observation, but is representative of a known object or place, then the AI might “guess” or “retrieve” what is to be drawn or is in the process of being drawn and attempt to correct the artist's work in progress. One might think of this *corrective drawing* in the spirit of spell-checking or grammar checking in word processing systems.

Often the discussion led to a point where the idea of a *predictive* element of a drawing AI was mentioned. In this mode, the AI attempts to guess the content that is being drawn, predicts aspects of or a version of the drawing, and presents portions of or the full drawing to the artist. Different scales of prediction are possible, such as the next stroke, or a fully completed drawing. An AI can not only predict and/or make suggestions without prompting from the artist, but also respond directly to the artist, for instance, who might want to know what their drawing “looks like” to the AI and might adjust their drawing accordingly. In addition, some aspects of these predictive uses of AI were proposed as a potential *labor-saving mechanism* for the artist. For example, some aspects of drawing is very tedious,especially when it comes to the texturing phase. An artist might be manually filling in a specific texture when exhaustion sets in, leading to deviation from a consistent style. The notion of having an AI complete the task was received favorably.

*Scene completion* is another, related, form of collaboration that an AI is thought to be able to fill. For instance, some artists work on drawing a character or a portrait, and they would like an idea as to how to fill in a background that makes “sense.” In this case this is partially predictive, but also there is an open element here for an AI to be creative in how it might fill in a background or accompanying accessories for the drawing. In this sense, an AI would need to be able to guess the context of the setting that the drawing is within.

An AI might also help to address *artist's block*. This was expressed as a common theme amongst the full-time artists, who draw frequently. In fact, one of the things that they often want to know is if what they are drawing is stereotypical of their own work. In other words, artists might desire novelty in their work, but often rely (subconsciously or otherwise) on habits or “go to” tropes in moments of conceptual difficulty. The problem of deciding what to draw for an artist could be viewed in terms of a form of *exploration-exploitation* trade-off, where the artist is either exploiting their knowledge (i.e., drawing the same thing that has worked in the past) or exploring new directions (i.e., trying to draw something that is novel to them). An AI might be able to contribute to the drawing or show some visuals that help an artist break out of these stale patterns. Often times, participants want to sit down and draw something new, but have a hard time thinking of what to draw. Having an AI that could aid in these moments is appealing to these artists, although the details of how the AI might actually do this was not discussed in depth.

To further expand on the concept of an AI as creative partner, the allegory of Microsoft's *Clippy* [i.e., the digital word processing assistant paperclip from Office '97 (Whitworth and Ahmad, 2006)] was brought up and contrasted with an improvisational accompanist in a jazz combo. Conceptually this was a dead end for our study participants, perhaps because of the lack of clear understanding of how the improvisational analogy translates to the visual arts. For musicians, a clear precedent exists for collaboration in the form of “jamming” together; however in visual arts, this concept is not so clear. This also might be because almost all of the participants' practices were representational drawing, as opposed to free or automatic drawing.

#### 4.2.3. Interview Summary: Key Themes

We applied theoretical thematic analysis (Braun and Clarke, 2006) to the transcripts from the semi-structured interviews. We identified three key themes:

1. **Drawing with physical mediums is a traditional and primary way of creation for visual artists**. Participants expressed clear benefits with respect to drawing with physical art media (e.g., pen, pencil, paint, paper, canvas). Physical surfaces feel immediate and direct (e.g., drawing with a pencil and then being able to smudge drawn lines with a finger). Paper provides a tactile response through friction with the drawing tool. This contrasts to the feeling of working with a digital drawing tablet, which has the haptic response of pushing a piece of plastic upon glass.
  “Pen on glass. I just don't think that it bears. I actually really love—I actually really like doing that today. And I chose this pencil [*referring to a pencil used earlier in a drawing exercise*] because it's chalky it's got sort of—it's got friction. And I like that. Otherwise you can kinda—you can just skid off. Like to actually stop and start. Like I've worked on glass with pens, you've got no stop and no start. You have to be quite definite about where you stop on the page of glass. Whereas, when you're working on surfaces, that surface helps you to sort of slow down and speed up. Does that make sense?”
  Even if the final product developed from our current prototype design were to take on a digital form, sketching with pencil and paper often are the initial steps toward embarking on a creative project. Digitizing a physical drawing typically occurs once during a project, via scanning or taking a photo with one's phone, as the effort is high to switch between physical and digital drawing tools. Once digitized, artists reported taking *more time* working on a piece because of the infinite possibilities provided by digital editing.
  “But then actually, I realized. it's actually more time consuming for me to do things digitally. Because I tend to refine things a lot. So if I get a chance to refine it. and there's always something imperfect when I do it digitally. It's like, I could edit it forever. While instead. traditionally sometimes I'll just leave it at that. Or, I'll just edit it slightly here. If I'm drawing something more like colorful or more complicated then I don't bother to scan it. Or I have to go to school to scan it if it's over A4.”
2. **Views on AI varied, where co-creative AI is preferable to didactic AI**. When presented with the idea of having an AI collaborator, artists and illustrators expressed a few reservations. They were concerned with something obstructing the direct action of drawing, being stressed by having an observer of their drawing process (even if artificial) and being annoyed at the premise of something instructing them in what to draw. Creative autonomy is important to the artist, and having something intervene within the drawing process is seen as distracting and undesirable.
  (*On a collaborative AI providing drawing suggestions*) “No I don't like it. I don't want suggestions. Like people guessing what you are doing, so I don't like it.”
  “I feel like for me, I don't know if I would use something like that because that's also because I have been drawing for so long, so I am used to my process or whatever.”
  On the other hand, artists were open to idea of having an inspirational agent, or muse, to contribute toward their idea process. Sometimes, coming up with ideas of what to draw is difficult, especially for those who draw on a regular basis. The study participants expressed interest in ways an AI might help them overcome “artist's block” via suggestive or inspirational prompts.
  “That would be interesting if it's something that's randomized your process. So for example, if you're always drawing that thing you have in mind, then it would be fun if something sort of like, messes it up for good.”
  In addition to exposing the artist to more variety, and creative AI was also seen as a potential time saving device.
  [Full-time illustration student] “Because we were speaking about AI, I feel it would be really cool if your tablet and pen could actually learn your patterns as an illustrator. And then when it senses that you're going to draw something, it will be like, ‘Are you trying to draw this?' And then you're like, ‘yes.' And then it just sort of, based on your usual. Because I feel when we draw faces or something, there's not that much variety. Especially when you draw from your imagination. And then you can just add the edits that you want. But it would be really cool if my tablet could do that for me. It was saved me so much time. And it's still like I drew it. Because basically just it put in there based on all the drawings I've done before.”
3. **Artists have a critical and skeptical view on automation of creative work**. Digital drawing tools have already impacted the working practise for artists. Full-time professionals describe how work has changed with the adoption of digital creative tools, such as Photoshop and high resolution digital printing.
  [Full-time professional] “It's quite sweet when you realise there's something it can do for you, and it can do it in tenth of the time that you do it in. So, in a way, I guess with Photoshop as well it's sort of. There is a bad side to it all. It has basically screwed my career in a sort of way. Because illustrators like me used to be really busy the whole time. Twenty-odd years ago, thirty years ago. Cause we'd have to be paid to do everything. And now, a lot of people do things themselves, cause they think ‘oh we've got Photoshop, we can do this. we've got clip art’…”
  Some illustrators are aware of the outputs of the computational creativity community with creative AI and advances, such as Google's *Inceptionism* (Mordvintsev et al., 2015). However, artists also share a critical view that these systems are indeed creative in the origination of ideas.
  “I see a lot of imitation [*in the output of creative AI systems*]. something comes out of it, which is cool. sure, it's like still unique in its own way, but it's not. I can't see how they [*AI systems*] were thinking. Or besides the fact that it was really intricately, like spiral drawings that they do. But, then they are probably, using some really crazy mathematical equation or something. But that doesn't spark anything. I'm just like, okay, it's a cool aesthetic. Like it looks really cool, because that's what they did to get to that point. But there was no major concept behind it. There's no reason why they [*AI systems*] did it.”
  Finally, some professional artists who participated in the study expressed some reservations about contributing to research, such as this study, that could lead to a produce that might eventually threaten their ability to find work as a creative practitioner in the future.

## 5. Objective Results: Drawing Exercises

In this section, we discuss the results and objective analysis of the middle component of the study: the drawing exercises. During each session, we asked participants to engage in three different drawing exercises, which we video-recorded. Our purpose was to observe how participants approach different types of drawing tasks (as outlined in section 3.4):

- **Observation**—“*Draw a still-life”*
- **Recollection**—“*Draw a bicycle from memory”*
- **Imagination**—“*Draw anything”*

Participants were prompted as above for each exercise and asked to complete each drawing within 10 min. The exercises were assigned in the same order (i.e., A, B, C above) for all participants. Due to the exploratory nature of the study, the 10-min drawing time was not strictly enforced, and some participants squeezed in extra finishing touches to their work after the “time's up” prompt was given. For others, the 10-min drawing time was considerably shorter than what they are used to, while others were able to complete the drawing within the allocated exercise drawing time. We labeled the completion status for each drawing as: *early* if they completed their drawing before the 10-min time limit; *done* if they completed their drawing at the 10-min limit or just beyond; and *midway* if they hadn't completed their drawing at the 10-min time limit and did not take extra time to finish.

**Figure 7:**
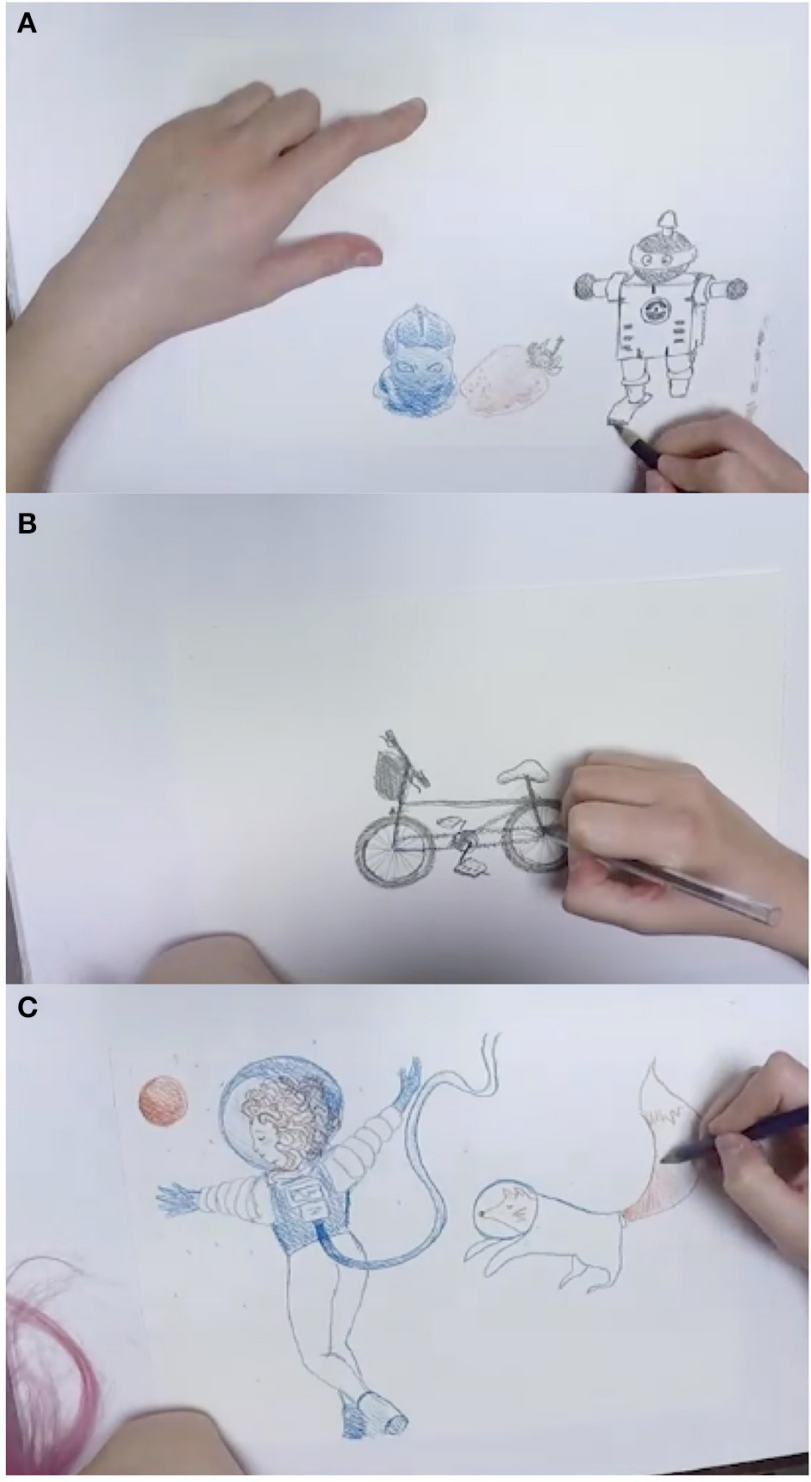
Drawing exercises in progress for a single study participant, showing **(A)** still life drawing, **(B)** drawing from mental image, and **(C)** free drawing.

An example of the sketches produced from the three drawing exercises for a single participant is shown in Figure 7. Collectively, the outputs from the drawing exercises comprise a small video gallery that can be viewed side-by-side in various combinations in order to contrast drawing styles across participant types. Each exercise provides a way to see how an individual begins the drawing task, blocking out space, drawing outlines, adding texture and detail. A sped up video showing side-by-side comparisons of different participants types is available as part of this publication^4^.

We analysed the drawing exercise video data by examining a range of statistics that describe the time spent on each exercise and characteristics of participants' drawing habits that could be discerned manually. This method was applied to the 63 drawing exercise videos^5^ and the results are discussed in section 5.1.

Although not applied directly here, we also considered classic *image processing* techniques for in-depth analysis of video features. Section 5.2 references an exploratory exercise which was conducted on a similar data set (a small sample gathered during pilot testing prior to the user study detailed here) and explains how lessons learned from this exercise could contribute to future automated analysis of drawing video.

### 5.1. Statistical Analysis of the Drawing Videos

Here we examine various statistics descriptive of the drawing exercises. These include: the time taken for drawing, ‘handedness” of participants, numbers of pages used per drawing, paper movement, drawing media, and completion status.

#### 5.1.1. Drawing Time

Table 4 illustrates statistics related to the amount of time spent drawing for each exercise. Time is reported in minutes and seconds, with mean and standard deviation in the first row of the table. Although all participants were given the same time window (~10 min) for each exercise, we were interested to compare the time spent by each participant on each of the three categories of drawing: observation, recollection and imagination. Exercise B (recollection) took the shortest amount of time for most participants (13 or 62%). Exercise A (observation) took the longest amount of time for most participants (11 or 52%). We can loosely conclude that many participants found observation took longer while recollection was faster.

**Table 4:** Statistics on time spent drawing for each exercise.

	**A**	**B**	**C**	
	**Observation**	**Recollection**	**Imagination**	**Total**
Mean	10:33 (01:15)	08:37 (02:41)	10:08 (01:43)	29:18 (04:10)
Shortest	3	**13**	5	21
Longest	**11**	5	5	21

*The first row reports mean and standard deviation (in parenthesis) reported, in minutes and seconds. The second and third rows report the number of instances (out of N = 21) where each exercise took the shortest and longest, respectively, amount of time for a given participant. Bold indicates which of the exercises had the top number of instances for the shortest and longest duration*.

#### 5.1.2. Handedness

From the video, we observed whether participants are left-handed or right-handed with respect to the drawing activities. Approximately 90% of the population overall is right-handed (Peters et al., 2006). In contrast, 81% of the participants in our study are right-handed.

We further examined the timing statistics discussed above with respect to handedness, to determine if right-handed participants were generally faster or slower than left-handed participants; however, no such conclusion is found. So we cannot say anything about the existence of a relationship between handedness and drawing speed based on the data set we collected in this study. Correlations between handedness and other characteristics are considered, in turn, as each additional characteristic is discussed below.

#### 5.1.3. Number of Pages

Most participants only used one page for each of the three exercises, but 6 (29%) participants used more than one page for at least one of the exercises. Nobody used more than one page for all three exercises, so there was no detected consistency with respect to using more than one page. There was also no correlation between handedness and the number of pages used. One primary reason for using multiple pages was that some participants reserved one drawing per page, while others drew small multiples on the same page. For example, in exercise B, some participants had false starts while working out how to draw a bicycle and used multiple pages in the process. One participant worked on many ideas in exercise C, and iterated through many drawing very quickly.

#### 5.1.4. Paper Movement

The paper on which participants drew was placed on the table in a bounded region to ensure that it was visible by the video camera capturing the exercises. Initially, the paper was placed squarely on the table. Inevitably, the paper moves slightly as people draw. But some participants purposely turn the paper while they are drawing. They orient the drawing at a comfortable angle for a drawing operation, such as drawing a straight line, or filling in a space with a texture. Also, at moments in order to assess their work, some participants pull back and view their drawing from a different perspective. The majority of participants (18 or 86%) moved the paper when working on at least one of the exercises. Of these, seven participants moved the paper during all three exercises. Interestingly, all of the left-handed participants moved the paper during at least one exercise; thus all of the participants who did not move the paper are right-handed.

#### 5.1.5. Drawing Media

Participants were permitted to employ whatever drawing media they desired, and we recorded a range of different tools. These are: charcoal (plain, medium or stick), eraser, finger smudge, graphite (or graphite stick), marker (brush, chisel, posca, tombow brush, tombow felt, tombow fine), pastels, pen (ballpoint, gel, micron 0.03, micron 0.05, micron 0.1, micron 0.3, mitsubishi felt 0.05, mitsubishi felt 0.5, pilot felt, pilot uniball, stabilo fine, tombow brush), pencil (2B, 4B, color, HB, mechanical, prismacolor ebony) and watercolors. One participant employed a digital drawing tablet for the free-drawing exercise (exercise C), using an Apple Pencil stylus with the Procreate sketching application. Table 2 shows the distribution of drawing tool usage across all 21 participants. The most commonly used tools were some form of pencil (employed by 81% of participants), eraser (57%), and some form of pen (57%).

Next we look at the tool usage per artist. Most artists (19 or 90%) used more than one drawing tool for at least one of the drawing exercises. Nine artists (43%) always used more than one tool (i.e., for every drawing). Only two artists only used one tool for each drawing. Interestingly, one of them used the same tool for all three drawings (a 2B pencil), whereas the other used two different tools but did not change between tools during a drawing (prismacolor ebony pencil for exercises A and C, and graphite for exercise B). Again there was no correlation between handedness and tool usage.

#### 5.1.6. Completion Status

As described earlier, we labeled each drawing according to whether the participant finished drawing before the 10-min time limit (“early”), at or shortly after the time limit (“done”) or did not complete their drawing (“midway”). Table 5 shows the distribution of completion status labels with respect to each drawing exercise. For exercises A and C, most people completed their drawing at or shortly after the time limit. For exercise B, most people finished early; indeed, for exercise B, everyone finished. Overall, most people completed their drawings, as only 11 (17%) out of the total number of drawings (21 × 3 = 63) were labeled as incomplete.

**Table 5:** Completion status for each exercise.

	**A**	**B**	**C**	
	**Observation**	**Recollection**	**Imagination**	**Total**
Early	4 (19%)	**13** (62%)	4 (19%)	21 (33%)
Done	**10** (48%)	8 (38%)	**13** (62%)	31 (49%)
Midway	7 (33%)	0 (0%)	4 (19%)	11 (17%)

*Bold indicates the top completion status for each exercise*.

Looking at the completion rate per participant, we find that 12 (57%) of the participants completed all three drawings. There is no correlation between completion status and handedness, as half of the left-handed people completed all three drawings and half did not.

### 5.2. Future Opportunities Using Image Processing

The drawing videos provide a rich data set to which a range of automated *image processing* techniques could be applied in order to identify features that might provide insight into different artists' drawing styles. To inform this direction for future work, an exploratory exercise (Kim, 2019) was conducted in which a few *computer vision* and *machine learning* methodologies were applied to a small sample data set comprised of three drawing videos collected during pilot testing for the user study detailed in this article.

This exercise explored automatic identification of features from the sample videos, including: stroke length and stroke speed patterns; hovering habits (i.e., holding the drawing implement poised above the drawing surface); and paper usage, including the amount of a page typically used, the region of the page covered (e.g., top, bottom, middle, left, right portions) and the location pattern, such as starting in the middle and moving out or drawing from top to bottom or left to right. For example, referring to Figure 7, one can see that the lower right portion of the paper was utilized for drawing exercises A and B, whereas the entire page was utilized for exercise C.

In order to compute the features listed above, the first step is to detect the location (coordinates) of the endpoint of the drawing implement within each video frame, as well as the length of line(s) drawn between one frame and the next. For example, the endpoint can be seen clearly in Figures 7A,C but is obscured by the artists' hand in Figure 7B.

The exploratory exercise attempted to automatically classify each video frame as either “hidden” or “not hidden,” indicating whether the endpoint was clearly visible or not. Further, each “not hidden” frame was classified as either “drawing” or “not drawing,” indicating whether the endpoint of the drawing implement was in contact with the surface of the paper or not (e.g., hovering above the page). These binary classification results, obtained by applying a sequence of classic image processing methods (e.g., edge detection and color segmentation to identify the artist's hand, drawing implement and the drawing artifact itself) followed by comparing various supervised learning algorithms (e.g., decision trees, logistic regression), proved to be slightly more accurate than random (between 50 and 60%) at predicting the correct labels for a given frame. However, a substantial amount of customized pre-processing of the data was required, in particular manually labeling enough video frames for training the supervised learning algorithms.

For frames classified as “not hidden” and “drawing,” it is then possible to compute the location of the endpoint of the drawing implement. This can be interpolated to an (*x, y*) coordinate within imagined axes that run along the edges of the drawing surface (page). Depending on where the endpoint is, some skew in this coordinate system may occur in the video frame; and this skew needs to be eliminated in order to reduce the noise that arises in trying to correlate series of (*x, y*) coordinates in consecutive video frames. Similarly, for consecutive frames classified as “not hidden” and “drawing,” the change in (*x, y*) coordinates can be mapped to interpolate a drawn line. An initial plan for finding the drawn line by calculating the difference between two consecutive images proved to be too noisy, largely due to the movement of the artist's hand. For example, even if the artist does not draw from one frame to the next—e.g., they lift their hand and drawing implement above the page—the shift in hand can obscure the line just drawn and confuse the drawn line detection method.

This exploratory exercise revealed a number of challenges that would have to be resolved before automated analysis of drawing video could be effective: (1) the raw video frames need to be cropped to remove noisy regions beyond the borders of the paper on which the human participant is drawing (e.g., around the edge); (2) many frames need to be labeled in order for an algorithm to learn to locate the endpoint of a drawing implement accurately within a video frame; (3) skew needs to be eliminated in calculating drawing implement endpoint accurately; and (4) significant sources of noise further confound automated video, including variable lighting conditions, occlusion of the drawn line by artist's hand and similar occlusion of the drawn line by the artist's drawing implement. The first two challenges involve manual processes that can be very time-consuming. Finally, the learned results obtained in this exploratory exercise were not very accurate, though better than random and may warrant further investigation.

## 6. Applying the Results

As mentioned in the Introduction (section 1), the outcomes from this user study contribute to the development of our prototype system for enabling AI-based collaboration in co-creative drawing practice. In this section, we identify a set of design requirements and a set of technical specifications for our prototype, generated from analysing the user study data. Finally, we describe our current demonstration system, explaining how the recommendations resulting from the user study are realized through technology.

### 6.1. Design Requirements for Co-creative Drawing System

The user study presented here suggests the following set of design requirements for our prototype system following directly from the thematic analysis described in section 4.2.3:

1. **Drawing Physically**. Artists employ a range of different types of drawing implements (see Table 2). We want our prototype to allow the artist to draw in a similar manner as was done in the user study, with their choice of physical media. The aim here is to maintain direct tangible interaction with the physical media as much as possible.
2. **Integrate Drawing Texture**. Video from the drawing exercises exhibited a varying range of textured outcomes from how an artist uses physical media (see section 5.1.4). We want our prototype to integrate the resulting texture from physical media into the way that the system observes the evolution of a drawing.
3. **Maintain Editorial Agency**. We want the artist to maintain primary editorial control with what is actually drawn (see section 4.2.2). This design criteria means that instead of having a drawing AI or robot modifying the art piece, the artist ultimately has the control as to what is actually drawn on the piece. Interactions with the drawing AI is more passive, where the artist is reacting to phenomena that the system presents as opposed to the system modifying the artwork itself.

### 6.2. Technical Specifications for the Research Prototype

We have identified a set of features for setting technical specifications that our prototype system should meet. These are listed and described below.

- **Spatial Resolution**. The spatial resolution of the input components dictates the fidelity the system is able to capture the drawn lines. For example, the study's video was recorded at a resolution of 1, 920 × 1, 440 pixels. Assuming, perfect framing of an A4 sheet of paper (297 × 210 mm), the resolution is 6.4–6.8 pixels/mm. However, in practise the drawing surface typically occupies about a 1/3–1/4 of the image, so this resolution is 2–3 pixels/mm. In contrast, a commercial drawing tablet captures a resolution of 100 points/mm.
- **Temporal Resolution**. Temporal resolution dictates how often the system can capture the incremental progress of the drawing process. This is a function of the data-capture frequencies of the input components. For instance, the video recordings of the drawing exercises occurred at 25 Hz, which from our initial image analysis (see section 5.2) provides a coarse capture of drawn lines. In contrast, commercial drawing tablets digitize pen positions at 200 Hz, which provides higher resolution detail of how lines are drawn. Another consideration with temporal resolution is the case where multiple input components are used. In this case, data captured at different frequencies will have to be correlated to each other temporally.
- **Baseline Responsiveness**. Physical media is as lively as physics allows. Because of this, the system should be as responsive as possible to physical drawing. There is a minimal latency for when the artist makes a mark and the system is able to respond on the drawing surface. A baseline response time of under 0.1 s is necessary for the sense of instantaneous reaction from the system (Nielsen, 1994).
- **AI Processing Time**. In addition to the baseline response time, the system's AI requires processing time. The amount of time the system has to process input and render an output dictates how sophisticated a response is possible. For instance the Javascript implementation of the *sketch-rnn* model (Ha and Eck, 2017) can process a generating vector drawings within 1/60-th of a second and maintain interactivity. There is trade-off between the speed and frequency of the system's response and the processing time allocated to the AI while remaining responsive.
- **Resilience**. Individual components will experience noise and disruption as part of their input processes. Occlusion of the drawing activity by the artist's body and by other objects placed on the drawing surface was a major issue identified in the analysis of the drawing exercises (see section 5.2). Lighting conditions impact the quality of image capture through shadows, flickering from light sources and reflections off the artist's body and accessories (e.g., eyeglasses, metal jewelery). While one can mitigate the setting in which the system operates in experimental lab conditions, for operation “in the wild” (e.g., the artist's studio), its components would require larger tolerances on the input data.
- **Endurance**. The research system would have to maintain the throughput and be able to manage the volume of data from a typical drawing session. At a minimum, the research system should be able to sustain operating through three 10-min drawing sessions that were executed as part of the pilot study. However, we know, from the survey (**Q9**, Figure 4), most artist's working time is between 1 and 2 h per session. The single camera from the drawing exercises recorded 1 Gb of video for every 4.5 min. Even if the video is processed, at the early stages of the development of the research system, one would anticipate storing high fidelity video of the drawing session for offline processing and training.

### 6.3. Technical Set-Up of Prototype System

**Figure 8:**
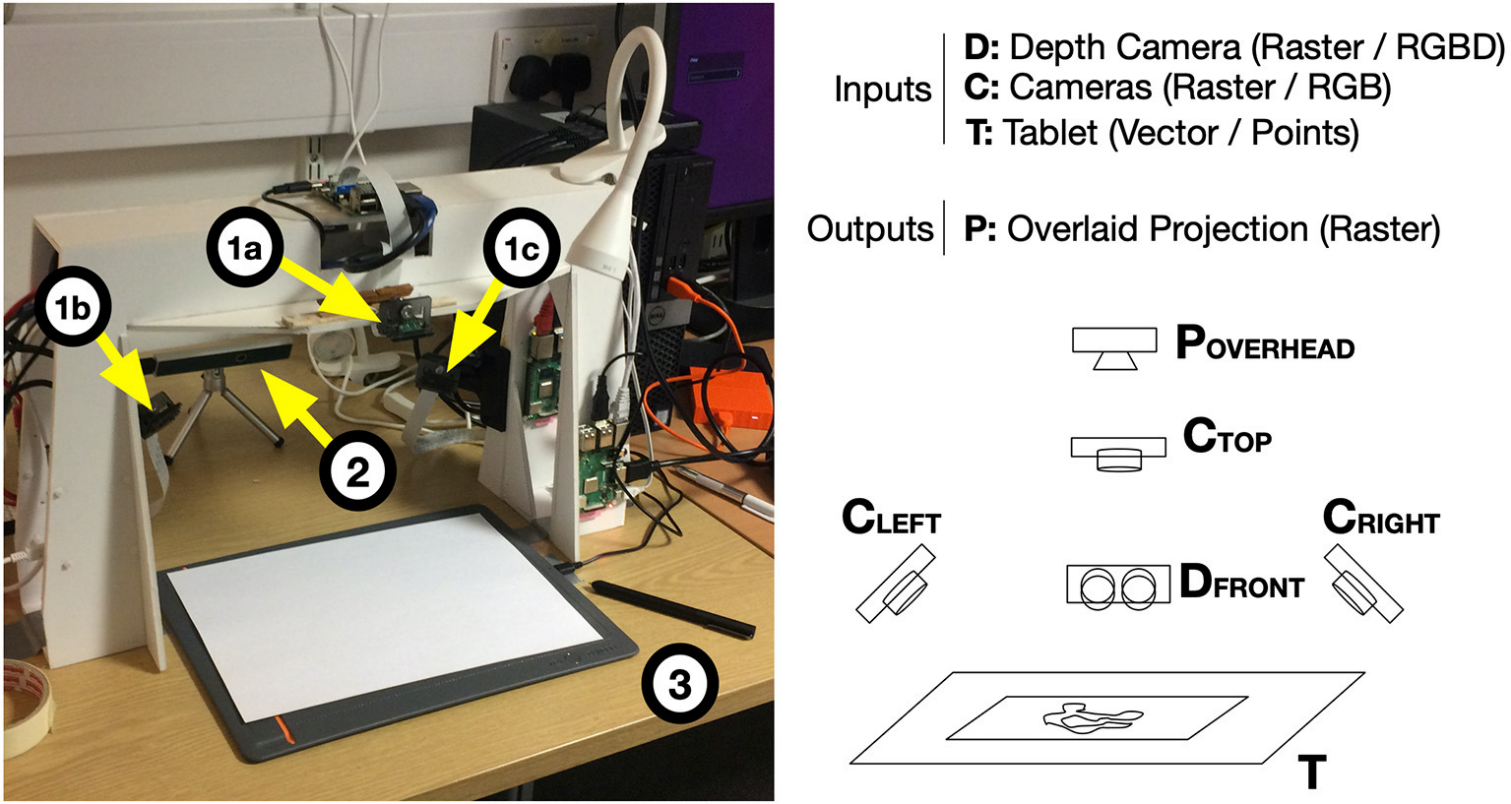
**(Left)** Design of prototype research system with Raspberry PI cameras **(1a–c)**, depth camera **(2)**, and WACOM Bamboo Slate digital “sketchpad” **(3)**, each with a dedicated Raspberry PI communicating via ROS (http://ros.org). **(Right)** Schematic for prototype research system.

With the technical considerations from section 6.2 in mind, we have developed an early version of our prototype system. Figure 8 shows an image of the prototype (*left*) and corresponding schematic design of its components (*right*). Each component is controlled by a dedicated Raspberry PI^6^ coordinated through a distributed messaging framework that is commonly used in robotics and autonomous systems research, namely the Robotic Operating System (ROS)^7^. The sensing components are: three Raspberry PI v2 cameras^8^ (**C**_TOP_, **C**_LEFT_ and **C**_RIGHT_), an Intel RealSense SR305 depth camera^9^ (**D**_FRONT_), and a WACOM Bamboo Slate^10^ digital “sketchpad” (**T**), which uses a pressure sensitive pen that tracks movement and produces marks on physical paper. The cameras observe the drawing area from multiple angles and record textural aspects of the drawing, while the digital sketchpad records a vector representation of the pen's movements.

Our design includes a projector **P**_**OVERHEAD**_ which overlays the robot's interaction upon the drawing surface and will be utilized in a future study. Through the use of projection, as the AI's only mode of output, the human artist maintains sole physical agency to manipulate the physical drawing in progress.

## 7. Discussion

This section discusses the results of the user study in light of related work (see section 2) and considers how might one categorize a collaborative drawing AI. Section 7.1 compares the outcomes from the user study in categorizing a drawing AI in terms of how it interacts with an artist. Section 7.2 discusses various actions that the AI might take in a co-creative drawing process and compares existing co-creative drawing systems from section 2.4 within this context. Section 7.3 looks at how the outcomes from the user study impact the ongoing design of our prototype system.

## 7.1. Interaction Factors

We identified a set of factors indicating how an AI might interact during the artist's process. Table 6 characterizes the related co-creative drawing systems in terms of the interaction factors and actions discussed in this section. They are:

1. **Synchronicity** *Synchronous* drawing means that the artist and the AI are taking turns drawing onto the piece one at a time. Turns may alternate, or one actor may take the *initiative* (see below) to take multiple turns in a row, without waiting for or requesting permission from the other. In synchronous mode, the notion of *turn* must be defined so that each actor can signal to the other that they have completed their turn; or turns could be based on fixed lengths of time (e.g., 5 min each). *Asynchronous* drawing is where the artist and the AI draw at any time, independently of each other. In this case, the AI and the artist may draw with varying initiative.
2. **Initiative** Initiative describes the level of autonomy given to the AI for interacting with the drawing. A system may have high initiative, in which case the AI will be more likely to contribute to the drawing on its own accord (i.e., without waiting for the artist to request assistance). In contrast, a low initiative AI may be required to wait for a prompt from the artist before it can contribute to the drawing. The level of initiative might be set by the artist, such as in the *DuetDraw* (Oh et al., 2018).
3. **Spatial overlay** This refers to where the artist and AI are drawing with respect to each other. Their drawing canvas may be *shared*. In this case their drawing interactions might *overlay* each other or occur in separate regions on the canvas from each other. This could be defined ahead of time by the artist or by the AI, or by some negotiation process before drawing starts. This could also be re-negotiated during the drawing process. The flexibility of this factor will be constrained by the physical limitations of the prototype system (e.g., the human artist can take their pen and go over to a random wall and start drawing, but the AI will only be able to sense and respond within the regions accessible to the system's cameras and projector).
4. **Actions** An AI will take one or more actions to collaborate with the artist. Through the user study and from reviewing related co-creative drawing systems we compiled a non-exhaustive list of these actions which are elaborated further in section 7.2.

**Table 6:** Co-creative drawing systems characterized by interaction factors (section 7.1) and actions (section 7.2).

**System**	**Initiative**	**Synchronicity**	**Actions**	**Spatial overlay**
*Exquisite Corpse* Brotchie and Gooding, 1991	Low, turn-taking	Synchronous	Completion	Shared canvas, no-overlay
*FluidSketches* Arvo and Novins, 2000	High, continuous	Asynchronous	Correction	Shared canvas, replaces drawing
*AutocompleteAnimation* Xing et al., 2015	Low, on request	Synchronous	Transformation	New drawing
*ShadowDraw* Lee et al., 2011	High, continuous	Synchronous	Suggestion	Shared canvas, overlay (underlayer)
*Drawing Apprentice* Davis et al., 2016a,b	Adjustable, continuous, turn-taking	Asynchronous, synchronous	Improvisation, trace, transformation, imitation	Shared canvas, overlay
*DuetDraw* Oh et al., 2018	Adjustable, continuous	Asynchronous	Completion, transformation (colorize)	Shared canvas, overlay
*Creative Sketching Partner* Karimi et al., 2019	Low, on request	Synchronous	Conceptual shift	Separate, adjacent canvas
*collabdraw* Fan et al., 2019	Low, turn-taking	Synchronous	Completion	Shared canvas, in-place
*DialogCanvasMachine* Cabannes et al., 2019	Low, turn-taking	Synchronous	Suggestion	Shared canvas, overlay
*D.O.U.G*. Chung, 2015	High, continuous	Asynchronous	Imitation	Shared canvas, overlay
*ArtTherapyRobot* Cooney and Menezes, 2018; Cooney and Berck, 2019	Low, turn-taking	Synchronous	Imitation, visual metaphor	Shared and separate canvases, overlay

### 7.2. AI Actions

In this section, we describe a range of actions that AI might take while an artist is drawing. These are based on an analysis of the works described in section 2 and the themes from the artists' interviews (section 4.2).

#### 7.2.1. Correction

An AI might *correct* the artist's drawing according to specific model of drawing. For instance, the *FluidSketches* system (Arvo and Novins, 2000) identifies drawing primitives in the artist's drawing and converts them to idealized geometric primitives. This form of AI action was a common theme that came up in the interviews, and often the artists saw this as something useful for learners but not necessarily for themselves (see Key Theme 2, in section 4.2.3).

#### 7.2.2. Tracing

An AI might *trace* over what the artist is drawing, which is a strategy in the *Drawing Apprentice* system (Davis et al., 2016a,b). Conversely, an artist might utilize tracing as feedback to the drawing AI, reinforcing the drawn strokes as desirable, or to signal a correction to the AI. The concept of the tracing action did not come up in the artists' interviews. However, what did surface was a desire for digital art programs (e.g., Photoshop) to perform better *vectorization*, or converting a raster scanned image into discrete vector art.

#### 7.2.3. Imitation

A less strict strategy to tracing is to *imitate* what the artist is drawing. This can occur both on the same canvas, as with the *D.O.U.G*. robotic drawing system (Chung, 2015), or on a separate canvas as with the *ArtTherapyRobot* (Cooney and Menezes, 2018). In the interviews, imitation did not come up as a possible AI action. However, there was a concern as to whether the artist has the copyright with respect to AI generated imagery.

#### 7.2.4. Suggestion

An AI might *suggest* to the artist what to draw next based on its model of the artist's drawing process. An AI which operates like Microsoft's *Clippy* digital assistant would suggest something for the artist to draw, and the artist would approve or reject the suggestion. Instead of seeking approval, the AI could be continually suggesting something to draw, in the manner of *auto-complete* predictive text interactions. The *DialogCanvasMachine* (Cabannes et al., 2019) suggests drawn strokes in the form of a projection onto a physical canvas. The artist draws their interpretation of the projected drawing onto the canvas. The *ShadowDraw* (Lee et al., 2011), instead of explicit drawing, displays gradient of drawings suggestive scaffolding which the artist can draw over. Suggestion of what to draw, as a result of the AI predicting what the artist might draw next, was a strong theme in the interviews. Creative autonomy was important for the artists, and some saw having the AI suggest the next stroke as getting in between them and their drawing (again, see Key Theme 2, in section 4.2.3).

#### 7.2.5. Improvisation

An AI might be like an *improvisational* partner contributing to a drawing according to its own drawing process without suggesting anything to the artist. This improvisation could be reactive to what the artist is drawing, such as with the *Drawing Apprentice* system (Davis et al., 2016a,b). Improvisational actions taken by the AI were proposed as a concept by the interviewer, however conceptually it was difficult for artists to see how *visual* collaboration might occur. One artist discussed their use of improvised drawing games as part of a workshop for drawing for children (e.g., each person taking turns adding a stroke to a composition).

#### 7.2.6. Completion

An AI might *complete* a drawing for the artist. This completion might be based on an explicit model that the AI uses of the drawing, such as the animal categories in the *collabdraw* system (Fan et al., 2019). It may attempt to identify and complete what the artist is drawing as in the *DuetDraw* system (Oh et al., 2018). The *DuetDraw* system also allows the artist to have the AI *colorize* the drawing, which is a form of completion as well. The AI might complete the drawing according to its own model in the manner of the parlor game, *Exquisite Corpse* (Brotchie and Gooding, 1991), in which participants take turns to contribute to a drawing without visible knowledge of what the other person is drawing, producing a novel surrealist outcome. Completion did come up as a theme in the interviews, in particular as a labor saving device for completing aspects of a composition, such as background rendering or texturing (see discussion in section 4.2.2).

#### 7.2.7. Transformation

An AI might *transform* what the artist has drawn in a different style, which is used in the *Drawing Apprentice* (Davis et al., 2016a,b) and the *DuetDraw* (Oh et al., 2018) systems. Transformation assumes a form of replacement of the drawing in contrast to completion which is additive to the drawing. In the *AutocompleteAnimation* system (Xing et al., 2015), the AI produces the next frame in an animation series based on previous drawn frames. One might think of this process as transformation of a drawing into its consecutive frame. This concept of automating this *tweening* process did arise in a discussion for the labor-saving contributions of an AI collaborator.

#### 7.2.8. Conceptual Shift

An AI might inspire an artist by providing a *conceptual shift* in what they are drawing. The *Creative Sketching Partner* (Karimi et al., 2019) displays reference imagery that is visually similar but semantically is different to what an artist is drawing. In this case the AI is not contributing drawn strokes to the artist work, but is showing a reference. This concept of having the AI evaluate what the artist is drawing and searching for similarity within some knowledge base did come up in the interviews. In particular, there was a desire to see how novel what one is drawing, and to have the AI evaluate the originality of the work.

#### 7.2.9. Visual Metaphor

An AI might contribute to that artist's drawing based on a *visual metaphor* of a sensed emotional state in the artist, which is utilized by the *ArtTherapyRobot* (Cooney and Berck, 2019). This theme of visual metaphor and sensing the emotional state of the artist did not come up in the interviews.

### 7.3. Impact on the Design

In this section we discuss open questions which arose as a result of the user study and how they impact the development of the prototype system.

#### 7.3.1. How Can the AI Reason About Spatial Aspects of the Drawing Process?

There are two dynamic systems happening simultaneously which are related in the sense of where the drawing tools touch the surface.

First, there is the movement of the artist's body, their arm, their hand, the drawing tool, the tip of the tool as it approaches and touches down varying in pressure and movement, and leaves the drawing surface. There is a spatial strategy to how an artist draws. And the movements are different from how a machine, such as a pen plotter printer, would render an image. Robotic drawing systems, such as *The Painting Fool* (Colton, 2012), are programmed explicitly to follow an artist's style of movement as opposed to that of the plotter. Having a richer understanding of the dynamics of the artist's body can enrich the development of such systems.

Second, there is the evolution of the drawing artifact itself as a dynamic system. From our analysis of the drawing exercise videos, artists drew a lot but erased little. Most of the time the drawing was “additive,” which means that it grew with respect to spatial coverage of the drawing surface. Indeed, some erasures are additive in themselves, in the form of added marks, smudges, and/or smears on the surface.

Thus, we can also explore spatial analysis approaches to the evolution of the drawing. Measuring the spatial arrangement of points through *point-pattern analysis* and *density estimation* (de Smith et al., 2018) would provide information about “where” an artist is drawing and “when.” For instance, a heat map over time, indicating where on the page the “hot area” is (i.e., where the artist is currently drawing) vs. “colder” areas that are older in the drawing timeline, would provide user information for a co-creative partner to either avoid disturbing the artist by drawing in the same area or to intervene where the artist is actively drawing.

#### 7.3.2. How Might Artist Fatigue Influence System Interaction?

Fatigue is real, for artists, but not so for machines, at least at the scale of a drawing session. Drawing is a physical activity, even on digital devices. There is a warm-up period, a period of performance and then onset of fatigue. It may be possible to measure this physical cycle within the dynamics of the artist drawing, and have the co-creative AI consider and respond to fatigue within a drawing. With the exception of the *ArtTherapyRobot* (Cooney and Berck, 2019), which models the emotional state of the artist, the existing co-creative drawing systems are “robotic” and not empathetic (i.e., generally not responsive to changes in the user's behavior).

#### 7.3.3. How Does the Drawing Medium Impact Digital Image Representation?

Drawing acquisition from a camera input is a research challenge. Cleanly acquiring the drawing, segmenting it from the background of the drawing surface and converting it into a vector representation is a research challenge. Existing systems that work with physical media utilize bold painted lines, such as the *DialogCanvasMachine* (Cabannes et al., 2019), to produce a high contrast image. From our survey, pencil is the most common medium for drawing, and may leave very light marks on paper which may be difficult for a camera to pick up. However, another outcome from the survey was that the physical-to-digital workflow is more typically from a drawn image on paper into Photoshop, and the artist would work on drawing at the pixel level. Only when they required scalable or crisp line-work, did the artist vectorize their drawings. If the novelty of drawing with analog media is maintaining a rich texture, then a co-creative drawing system might work at the pixel-level instead of initially vectorizing the drawn input. Such a trade-off would require the prototype research system to have a richer representation of the drawing, rather than the common vector points-and-strokes object model.

#### 7.3.4. How Could an AI Interact With an Artist?

Having the AI interact with the drawing surface that is clear to the artist is an interface challenge. The AI could draw or have their interactions presented on a separate canvas or screen. However, this loses the immediacy that having the artist and the AI share a common drawing surface. One of our primary design requirements (see section 6.1) is to allow the artist to draw upon a physical surface. In this case, the AI could have a robot drawing on the surface, like in the *D.O.U.G*. system (Chung, 2015). Another design requirement is maintaining editorial control, so having the AI drawing physically as well is not practical. In this case there are two manners in which the AI could interact with the drawing.

First, it could use projection, as is done with the *DialogCanvasMachine* (Cabannes et al., 2019). Projection is non-destructive to the drawing, as opposed to having a robot draw upon the surface as well. Another advantage is that projection allows a large variation in options for drawing surfaces. Thicker materials, such as canvas, boards, and walls are eligible surfaces in this sense. However, there are trade-offs with projection. One primary obstacle is that occlusion of objects in front of the projection casts shadows onto the surface. Shadows cause sharp contrasting shapes upon the surface which and that may confuse camera input of the drawing. Projection also is sensitive to lighting conditions. Many participants desire drawing in brighter lighting conditions with natural light. A projection could provide more of this lighting. However, the contrast of the projected imagery risks being washed out under bright lights.

An alternative method is to project underneath the drawing surface. This is similar to the light-box set-up wherein a light source located underneath the drawing surface shines upwards and allows the artist to trace imagery onto thin media, such as light weighted paper. In this case, the paper would need to be thin enough to project through. Such an effect could also be produced through the use of a strong (bright and high-resolution) monitor as the drawing surface (e.g., a digital display table).

#### 7.3.5. How Could a Co-creative AI-Based Drawing System Introduce Artists to Collaborative Drawing?

Collaborative or collective sketching activities were rare amongst most participants of our user study, especially amongst part-time and students (see sections 4.1.2 and 4.2.2). In addition, most people interpreted “collaborative drawing” as attending life-drawing classes and looking at each other's drawings. Only one participant described having participated in a collaborative drawing activity where more than one individual drew on the same piece of work. This lack of collaborative drawing experience might be a novel opening for them working with a collaborative drawing system, with less preconceived notions toward collaborative drawing. In fact, the interview discussion in the study might have had an impact on the artist's practise in opening their eyes to collaborative drawing practice. Finally, it is likely for future participants that using our research prototype might be their first collaborative drawing experience

## 8. Summary and Conclusion

This article has presented the results of a user study of drawing practitioners, conducted with the objectives of understanding their drawing practises and workflow and discussing their thoughts on collaborative drawing with an AI-supported system. The study gathered survey data, videos of drawing exercises and transcripts of interviews discussing artists' working habits and ideas around a co-creative drawing AI. Having the analysed the data, we have identified some key themes, design criteria and technical specifications for a prototype co-creative drawing system, and then presented a technical set-up of our current demonstration version of the system. We connected our analysis to related work in the literature, suggesting potential activities and characteristics for a co-creative drawing AI.

In closing, we mention lessons learned through developing, delivering, and analyzing the user study (section 8.1) and identify possible avenues for future work to come out of this study (section 8.2).

### 8.1. Lessons Learned

This section highlights a few of the key lessons we learned about the design of our user study from its delivery and analysis of the data gathered.

#### 8.1.1. Survey

The survey could have been improved to better extract specifics about an artist's drawing practise. The participants are overall passionate about drawing, so to ask “How long have you been drawing?” often just has them expressing their age, or an answer to the effect of “I've been drawing my entire life.” However, we were seeking something more specific about how much experience they had with drawing at a more serious capacity. There might be more specific ways of asking about experience than just asking for the number of years that they have been drawing.

Some participants struggled to characterize their typical working session. In particular, they found it difficult to assess how long a working session is, as there is variation in the time they spent working. This might be best broken up into a few questions. For example, we could have asked: “What is the shortest, the longest and the typical duration of a working session for you (or that you would consider a working session)?” Or, we could also have asked directly: “How do you define a working session?”

The survey also presented a laundry list of qualities of artists' work environments (**Q12**, see section 4.1.5). Some of these were related to each other in that they expressed opposing qualities (e.g., “Noisy environment” vs. “Quiet Environment”) while others were unrelated. We could have grouped these environmental qualities into more specific questions with ranges that inquire directly about factors, such as noise level, light level, public vs. private, and solitary vs. shared settings. Also, the context for this question could have been expressed more clearly as it was ambiguous whether we were referring to their *current* drawing environment or their *desired* drawing environment.

#### 8.1.2. Drawing Exercises

The video capture of the drawing exercises was very informative with regards to technical challenges of capturing image data of the drawing process. Lighting conditions in an academic office environment are generally poor for studio work. Fluorescent lights caused undesirable flickering. For the first half of the participant settings, the camera capture frequency was incorrectly configured (i.e., set to NTSC where the setting should have been PAL).

While the top-down egocentric camera view gives the best overview of the drawing area, much of the drawing actions were blocked by occlusion (see section 5.2). Given the choice of having only one camera, either a top-down from the side (opposite the handedness) or oblique from the top of the drawing surface would have captured more of the drawing actions.

There's a balance between wanting to set up the drawing environment so as not to distract the artist (i.e., a “natural” setting) vs. aiding in the technology-based capture of the artist at work. We allowed the artist to rearrange the drawing environment to suit their needs. However, having a consistently positioned drawing surface by affixing paper with tape would aid in the post-processing for analysis. In addition, when switching pages, moving the old drawing off camera and presenting a new clear surface would help with analysis, as some of the participants left previous drawings on top of the new drawing surface. But, given that the aim of this study was to understand how artists draw, adding more constraints would have limited our ability to capture the variables that a system deployed “in the wild” would have to accommodate.

#### 8.1.3. Interview Discussion

The interview discussions varied greatly in length despite planning the time for discussion to 30 min. Typical interviews lasted 1 h, and most of the time was spent on the first topic of their drawing practise. Artists had much to say about their work. However, even with breaks, this was a long discussion, and by the time the topic of collaborating with an AI arrived, interview fatigue often had kicked in. Given that obtaining views from artists on this question was one of our primary goals of the study, a more direct structure would have been to present the concept of collaborating with an AI earlier in the interview, and then to have more in-depth discussion about their work in relation to what an AI might be able to contribute.

### 8.2. Future Work

The open questions presented in section 7.3 mentioned avenues of future work within the context of the design of a co-creative drawing system. Modeling the dynamic movement of the artist's body, applying spatial analysis techniques to the evolution of a drawing, creating empathy models for an AI to use to respond to fatigue as the artist works are areas of research in enriching an AI's understanding of the drawing process. Improving the drawing acquisition workflow of physical mediums, such as pencil drawing, is another area of possible computer vision research area which might make an impact on artists' physical-to-digital conversion workflow. Through multiple cameras or an active camera mounted on a robot arm, higher resolution and more detailed images of the drawing surface are possible. Another area of research is how the AI's drawing is displayed onto the drawing surface. Similarly to the camera set-up mentioned previously, utilizing multiple projectors or a robot-mounted projector might improve the image quality of the projected overlay over the drawing surface. Most of the artists interviewed did not have any experience with collaborative drawing (in the sense we define it for our co-creative AI system). One direction for future work would be to conduct a follow-up study in which we specifically seek out participants who have experience with collaborative drawing and elicit information from them to influence the design of our prototype.

Our next steps with this line of research involve completion of our prototype system, expanding on the demonstration described in section 6.3, constructing and integrating computational models of artists' drawing processes based on some of the quantitative metrics discussed in sections 4 and 5, and conducting a follow-up user study to evaluate the efficacy of our prototype system.

## Data Availability Statement

The datasets presented in this article are not readily available because ethical clearance was given only to publish the research outcomes for the user study. Requests to access the datasets should be directed to CJ, chipp.jansen@kcl.ac.uk.

## Ethics Statement

The studies involving human participants were reviewed and approved by Research Ethics Office of King's College London as Low Risk Research, approved by the university's Biomedical & Health Sciences, Dentistry, Medicine and Natural & Mathematical Sciences Research Ethics Subcommittee. The patients/participants provided their written informed consent to participate in this study. Written informed consent was obtained from the individual(s) for the publication of any potentially identifiable images or data included in this article.

## Author Contributions

Both authors contributed toward the design of the user study, as well as writing and approving this work for publications. CJ conducted the user study, interviews, and collected the results for analysis and conducted the rest of the analysis present in this paper. ES conducted the statistical analysis of the drawing videos.

## Conflict of Interest

The authors declare that the research was conducted in the absence of any commercial or financial relationships that could be construed as a potential conflict of interest.

## Footnotes

^1^https://robots.ieee.org/robots/baxter/.

^2^https://www.etsy.com.

^3^https://www.instagram.com.

^4^https://youtu.be/fsf8wskYZdg.

^5^21 participants × 3 exercises.

^6^https://www.raspberrypi.org.

^7^http://ros.org.

^8^https://www.raspberrypi.org/documentation/hardware/camera/.

^9^https://www.intelrealsense.com/depth-camera-sr305/.

^10^https://www.wacom.com/en-us/products/smartpads/bamboo-slate.
