# Supplementary material for: Exploring Co-creative Drawing Workflows
Source: Front Robot AI. 2021 May 14;8:577770. doi: 10.3389/frobt.2021.577770 (PMC8160468; doi:10.3389/frobt.2021.577770)
Supplement: Supplementary file 1 [file Data_Sheet_1.PDF]

Drawing Practises, Workflow and Technology Collaboration Study Questionnaire

This survey is part of a study to understand drawing practices and discover possible technology collaborations with artists. You will be asked some background questions on your drawing practies and your use of and attitude towards technology both in your day-to-day and in your creative work.

Background

Q1. How long have you been drawing as part of your creative or professional practise?

- ☐ Less than 1 year
- ☐ 1-5 years
- ☐ 5-10 years
- ☐ Longer than 10 years

Q2. Have you done any formal training for drawing? (Check all that apply)

- ☐ Drawing training as part of primary school or secondary school
- ☐ Drawing as part of A-level preparation (i.e. A-level Art)
- ☐ Drawing training as part of a Foundation Art Degree
- ☐ Some drawing modules as part of Undergraduate University-level Degree Course
- ☐ Dedicated Undergraduate University-level Drawing Course
- ☐ Some drawing training as part of Post-Graduate Degree Course
- ☐ Dedicated Post-Graduate Degree Course
- ☐ Continuing Education Drawing Courses consisting of multiple sessions (i.e. short courses, evening classes)
- ☐ Single-session drawing classes or workshops
- ☐ One-on-one drawing tutoring/lessons
- ☐ Drawing apprenticeship
- ☐ Other formal training, not listed above:

Q3. Do you participate in any drawing communities, collectives, groups, or drawing sessions (i.e. life drawing sessions)?

- ☐ Yes
- ☐ No

If so, please describe what you participate or have participated in:

Q4. Do you earn money from your drawing practise or do you utilise drawing as part of your profession?

- ☐ Yes
- ☐ No

If so, please describe how drawing fits within your professional practise:

Q5. Rate the extent to which you would describe your drawing as being...

	Extremely	Very	Moderately	Slightly	Not at all
**a.** ...**illustrative**?	☐	☐	☐	☐	☐
**b.** ...**abstract**?	☐	☐	☐	☐	☐
**c.** ...drawn from **real-life**? (rendering an object, setting or subject that you **directly** perceive)	☐	☐	☐	☐	☐
**d.** ...drawn from a personal **memory**? (rendering an object, setting or subject that you perceived in the **past**)	☐	☐	☐	☐	☐
**e.** ...drawn from **imagination**?	☐	☐	☐	☐	☐
**f.** ...an expression of **emotion**?	☐	☐	☐	☐	☐

Q6. How frequently do you **doodle** or make drawings while your attention is otherwise occupied (i.e. draw absentmindedly during a meeting in the margins of a piece of paper)?

- ☐ Extremely frequently
- ☐ Very frequently
- ☐ Moderately frequently
- ☐ Slightly frequently
- ☐ Not at all

Drawing Practise

Q7. Which drawing mediums do you most typically draw with? (Check all that apply)

- ☐ Ball-point pen
- ☐ Felt-tip pen
- ☐ Nib-tipped pen and ink
- ☐ Pencil
- ☐ Charcoal
- ☐ Pastel
- ☐ Water-colors, or aqueous drawing medium
- ☐ Chalk
- ☐ Other drawing materials not mentioned above:

Q8. When do you draw? Is there a regular time (i.e. in the morning, late at night) or routine (i.e. with a coffee, after a long walk) that you have with your drawing practise?

Q9. How long are your drawing sessions typically?

- ☐ Less than 15 minutes
- ☐ 10 minutes to 1 hour
- ☐ 1 to 2 hours
- ☐ 2 to 4 hours
- ☐ 4 to 8 hours
- ☐ Longer than 8 hours

Q10. How do you typically work?

- ☐ Uninterrupted without taking any breaks
- ☐ Occasionally take a break
- ☐ Typically take many breaks

Q11. How do you typically focus your work during your drawing sessions?

- ☐ Focus on a single project
- ☐ Switch between 2-3 projects in a session
- ☐ Switch between more than 3 projects in a session

Q12. What is your drawing environment like? (Check all the apply)

- ☐ Solitary, typically work alone.
- ☐ Shared work space, or work around people
- ☐ Private setting (e.g. a private studio)
- ☐ Public setting (e.g. in a cafe, with in a store front with a large public window)
- ☐ Bright environment, with natural lighting
- ☐ Bright environment, with artificial lighting
- ☐ Dim environment, with a few focused lights
- ☐ Listening to music, with headphones or speakers
- ☐ Noisy environment
- ☐ Quiet environment
- ☐ Very warm, or hot temperature environment
- ☐ Cold temperature environment
- ☐ Other factors your would like to share about your drawing environment:

Q13. Are there things about your drawing environment that you would want to change to make it a more ideal work setting?

Q14. Do you carry around a sketchbook or a portable drawing pad?

☐ Yes
☐ No

If so, please give examples of the kinds of things that you draw in it:

Q15. Do you practise collaborative or collective drawing with another person or persons?

☐ Yes
☐ No

If so, describe the collaboration or collective drawing process:

Technology

Q16. Which of the following technologies do you utilise on a regular basis? (Check all that apply)

- ☐ Desktop computer
- ☐ Laptop computer
- ☐ Printer
- ☐ Digital scanner
- ☐ Mobile cellular phone
- ☐ Portable music device (e.g. iPod)
- ☐ Smart phone (e.g. Android, iPhone)
- ☐ Camera on a mobile phone
- ☐ Digital camera other than a mobile phone (e.g. DLSR camera)
- ☐ E-readers or dedicated e-book devices (e.g. Kindle)
- ☐ Tablet device (e.g. iPad, Android Tablet)
- ☐ Voice-controlled personal assistant (e.g. Apple Siri, Alexa on an Amazon Echo)
- ☐ Smart home devices (e.g. Google Nest)
- ☐ Smart watch (e.g. Apple Watch)
- ☐ Personal fitness device (e.g. Fitbit)
- ☐ Home gaming console (e.g. Xbox, Playstation)
- ☐ Portable dedicated gaming device (e.g. Ninetendo Gameboy)
- ☐ VR headsets (e.g. Oculus Rift, Google Cardboard)
- ☐ Personal robot or flying drone (e.g. Roomba vacuum robot, DJI Drone)

Q17. Which of the following technologies do you utilise as part of your drawing practise? (Check all that apply)

- ☐ Desktop computer
- ☐ Laptop computer
- ☐ Drawing apps on your mobile phone
- ☐ Drawing apps on your tablet device
- ☐ Digital drawing pad connected to a computer (i.e. Wacom tablets, Wacom Cintiq)
- ☐ Smart pens with traditional paper (i.e. LiveScribe, Wacom Inkling, Wacom Bamboo)
- ☐ Adobe Illustrator
- ☐ Adobe Photoshop
- ☐ Overhead analog projector or light enlarger
- ☐ Digital projector
- ☐ Other technologies/software not mentioned above that you use for drawing:

Q18. Which of the following technologies do you utilise to capture, document, or archive your drawn work? (Check all that apply)

- ☐ Photocopier
- ☐ Photos with an analog film camera
- ☐ Photos with a digital camera
- ☐ Photos with camera on your mobile phone
- ☐ Digital scanner
- ☐ Videos with digital camera
- ☐ Other technologies not mentioned above that you utilise to capture, document, or archive your drawn work:

Q19. Which of the following ways do you use to share, distribute, or sell your drawings? (Check all that apply)

- ☐ Show or sell original drawings or prints of drawings in gallery, fair, or art venue
- ☐ Publish drawings in printed magazine
- ☐ Publish drawings in online magazine
- ☐ Publish drawings in printed book
- ☐ Publish drawings in eBook or for portable readers
- ☐ Maintain a portfolio of drawings on personal web-site
- ☐ Share digital version of drawings on social media (i.e. Twitter, Facebook, Instagram)
- ☐ Share digital version of drawings on online artist community sites (i.e. DeviantArt)
- ☐ Publish online videos of you drawing or your drawing process (i.e. YouTube, Vimeo)
- ☐ Sell your work via online market places (i.e. Etsy, eBay)
- ☐ My drawings are not shared, distributed, or sold.
- ☐ Other ways that you use to share, distirbute, sell your drawings:

Q20. How interested would you be to utilise more technology in your drawing practise?

- ☐ Extremely interested
- ☐ Very interested
- ☐ Moderately interested
- ☐ Slightly interested
- ☐ Not at all interested

Q21. Is there anything else that you would like to say or comment on?

THANK YOU!

This is the end of the survey. Please place it in the accompanying envelope and let the interviewer know that you are done.
